# Neurobiological subtypes in alcohol use disorder and their phenotypic and clinical profiles

**DOI:** 10.1038/s41380-026-03748-4

**Published:** 2026-07-11

**Authors:** Leyla R. Brucar, Eric Rawls, Anna Zilverstand

**Affiliations:** 1 Department of Psychiatry & Behavioral Sciences, University of Minnesota, Minneapolis, MN USA; 2 Department of Psychology, University of North Carolina Wilmington, Wilmington, NC USA; 3 Medical Discovery Team on Addiction, University of Minnesota, Minneapolis, MN USA

**Keywords:** Diagnostic markers, Addiction

## Abstract

Alcohol Use Disorder (AUD) is associated with vast clinical, behavioral, and neurobiological heterogeneity that hinder both the efficacy of current interventions and future treatment development. While recent efforts have focused on stratifying psychiatric disorders based on underlying neurobiological markers, such approaches remain largely unexplored in AUD. To address this, we empirically-derived neurobiological subtypes of AUD and evaluated their reproducibility and clinical relevance. Participants with complete resting-state functional MRI (rs-fMRI), phenotypic and clinical data from the Human Connectome Project were included (N = 668; 58% Female; 22% AUD [mild AUD: n = 109; moderate-severe AUD: n = 38], 40% Female within AUD). Neurobiological subtypes of AUD were identified using a semi-supervised clustering approach based on graph theory metrics derived from whole-brain rs-fMRI. Subtypes were extensively validated for reproducibility using resampling, permutation testing, and temporal stability, and for clinical relevance using comprehensive assessments of multi-modal behaviors and clinical characteristics. Two robust and distinct neurobiological subtypes of AUD with unique clinical profiles emerged. Subtype 1 showed greater sensory-motor integration, but lower frontoparietal/salience integration, with elevated externalizing behaviors. Subtype 2 demonstrated the opposite pattern: greater integration in frontoparietal, salience, and default-mode networks, and lower sensory-motor integration, characterized primarily by internalizing behaviors. Subtypes did not differ by AUD symptoms or use characteristics. Importantly, the subtype structure and phenotypic profiles remained robust when including AUD individuals with comorbid cannabis dependence, supporting generalizability and translational relevance to clinically comorbid populations. In summary, we identified neurobiological subtypes of AUD with distinct phenotypic and clinical profiles, capturing greater nuance than previous phenotypically-derived subgroups, while remaining interpretable and clinically meaningful. These findings provide a robust framework for understanding AUD’s underlying mechanisms and underscore their potential for a neurobiologically informed approach to nosology, treatment allocation, and personalized interventions in AUD.

## Introduction

Alcohol Use Disorder (AUD) is one of the most prevalent and deadly psychiatric disorders, affecting nearly 29 million people in the U.S. alone [1]. Globally, alcohol misuse causes approximately three million deaths annually and contributes to more than 5% of the global disease burden - making it a leading cause of preventable and premature death [2, 3]. Despite its toll, treatment rates remain critically low: in 2023 only 7.9% of individuals with AUD in the U.S. received treatment [1], and worldwide, just one in six access care [4]. Among those who receive treatment, current interventions have limited success [5, 6]. This lack of efficacy has been attributed, in part, to the ‘heterogeneity problem’ - which postulates that distinct causal mechanisms underlie psychiatric disorders in different subsets of individuals [7, 8].

AUD heterogeneity, evident across symptomatology, severity, comorbidities, treatment mechanisms, and outcomes, has long been recognized [9, 10]. These complexities hinder both current AUD treatment allocation - where standard “one size fits all” interventions fall short - and future treatment development [11], warranting improved characterization and stratification to better inform clinical evaluation, develop personalized treatments, and boost treatment efficacy. In response, data-driven subtyping in AUD, via the application of clustering methods [12], has aimed to parse this heterogeneity by identifying more homogenous subgroups, or subtypes. Early efforts largely focused on heterogeneity in behaviors and clinical symptoms (e.g., drinking patterns, severity), while more recent approaches have emphasized mechanistically-relevant features to better capture underlying processes and guide the development of personalized treatments that target them [13–16].

Neuroimaging provides a promising avenue for further advancing this work [8], offering a non-invasive tool for investigating the underlying brain structure and function implicated in AUD. Considerable progress has been made in characterizing the neurocircuitry of addiction [17–19], revealing widespread dysregulation across brain networks involved in incentive salience and habit formation, executive functioning, self-regulation, and negative emotionality [17–21]. Despite this, however, definitive neuroimaging biomarkers remain elusive. Findings from AUD resting-state and task-based functional Magnetic Resonance Imaging (fMRI) research are inconsistent [18, 20–22], with some describing AUD as a ‘disconnection syndrome’ [23], marked by widespread reductions in connectivity, while others suggest ‘dedifferentiation’, characterized by redundant, widespread hyperconnectivity [24]. These discrepancies have been hypothesized to stem from methodological variability and importantly, the limitations of dominant case-control paradigms, which assume homogeneity within groups [18, 20, 21, 25]. Critically, these approaches may mask meaningful neurobiological differences, hindering replicability, biomarker discovery, and clinical translation [7, 25–27].

To address this, neuroimaging-based subtyping (biotyping) has emerged as a powerful strategy. Unlike symptom-based approaches, these methods identify subtypes based on common neurobiological signatures, which may be more effective at capturing underlying biological heterogeneity critical for guiding interventions [6, 8, 28]. These approaches have shown considerable promise within and across psychiatric disorders [8], with pivotal work in depression demonstrating their capacity for improved, targeted, subtype-specific treatments [29, 30]. Biotyping, however, remains underexplored in addiction [6], with only one study to date examining neuroimaging-derived subtypes in alcohol misuse [31] - underscoring a crucial gap and opportunity for research.

To close this gap, the current study aims to characterize the neurobiological heterogeneity among individuals with AUD. We hypothesized that multiple, distinct subtypes exist that underly the neurobiological and clinical heterogeneity observed in AUD. To investigate this, we leveraged Heterogeneity through Discriminative Analysis (HYDRA), a semi-supervised clustering algorithm [32]. Most neuroimaging-based subtyping studies have used unsupervised methods (e.g., k-means) to cluster individuals within a clinical population, independent of controls, making them vulnerable to confounding factors like demographics or unrelated pathology [8, 26]. In contrast, recent semi-supervised approaches - like HYDRA - seek a more ‘pathology-oriented’ solution by clustering the deviating patterns between a fixed ‘reference’ (i.e., controls) and ‘target’ group (i.e., AUD), minimizing confounding variation (see Supplementary Fig. 1) [12, 26, 32]. As input to HYDRA, we used whole-brain graph theory metrics derived from resting-state fMRI, providing biologically meaningful indices of functional brain-network topology [33]. We incorporated rigorous validation to assess both subtype replicability and clinical relevance [8]. This included selecting the most reliable cluster solution and graph metric for HYDRA through statistical analysis, and extensively validating reproducibility and the clinical relevance of the derived subtypes using comprehensive phenotypic and clinical data. Additionally, we demonstrated that traditional case-control comparisons failed to uncover meaningful distinctions with the same data - highlighting the importance of capturing neurobiological heterogeneity and the potential of the derived subtypes to inform personalized medicine.

## Materials and methods

### Participants

We analyzed data from the S1200 release of the Human Connectome Project (HCP), which was collected in Missouri, United States between 2012–2015, and included a comprehensive battery of phenotypic and clinical assessments and neuroimaging [34]. Our primary analysis included participants with complete fMRI resting-state Scan-Day 1 (REST1), phenotypic and clinical data. Alcohol abuse, alcohol dependence and cannabis dependence were assessed by the Semi-Structured Assessment for the Genetics of Alcoholism (SSAGA) using DSM-IV criteria. Participants meeting at least two diagnostic criteria for either alcohol abuse or dependence were combined into the AUD group in accordance with DSM-V criteria, and those meeting zero criteria were assigned to Controls. Since cannabis abuse was not assessed, we refer to these participants as having “cannabis dependence” (not cannabis use disorder). After excluding participants with cannabis dependence (n = 90) for the initial analysis, the sample included 668 participants (58% Female, Table 1). Among them, 147 had AUD, including 109 with mild and 38 with moderate-to-severe AUD (40% Female within AUD; see Table 1**)**. The HCP consortium does not specify if and how biological sex or gender was defined; therefore, we use the term sex/gender throughout. Resting-state data obtained from Scan-Day 2 (REST2; n = 646; 57% female; AUD: n = 141; Table 1) were used for subtype replicability analysis (see Supplementary Methods for details). All participants provided informed consent, and the study was approved by the Washington University Institutional Review Board (IRB #201204036) in accordance with the Declaration of Helsinki.

**Table 1:** Sample Demographics: Scan Day 1 and Scan Day 2.

Characteristic	Scan Day 1: AUD (n = 147)	Scan Day 1: Controls (n = 521)	Significance *(p-value*)	Scan Day 2: AUD (n = 141)	Scan Day 2: Controls (n = 505)	Significance (*p-value*)
**Age, mean (SD), years**	28.82 (3.52)	28.99 (3.77)	0.34	28.60 (3.28)	28.98 (3.79)	0.28
**Sex/gender, n (%)**						
Male	88 (59.86)	193 (37.04)	***<0.001***	85 (60.30)	190 (37.60)	**<0.001**
Female	59 (40.14)	328 (62.95)		55 (39.70)	315 (62.40)	
**Race, n (%)**						
Asian	8 (5.44)	41 (7.87)	0.05	8 (5.67)	40 (7.92)	0.05
Black/AA	12 (8.16)	80 (15.35)		11 (7.80)	78 (15.45)	
Caucasian	117 (79.59)	384 (73.70)		112 (79.43)	371 (73.47)	
First Nations	0 (0)	1 (0.19)		0 (0)	1 (0.20)	
Multiracial	4 (4.08)	5 (0.96)		4 (2.84)	5 (0.99)	
Unknown	6 (2.72)	10 (1.92)		6 (4.26)	10 (1.98)	
**Education, mean (SD), years**	15.33 (1.49)	14.99 (1.75)	0.05	15.31 (1.51)	15.00 (1.75)	0.05
**Income, $**						
<50,000	73 (49.66)	245 (47.02)	0.34	72 (51.06)	240 (47.52)	0.37
≤50,000 and <100,000	47 (31.97)	198 (38.00)		44 (31.21)	189 (37.43)	
≥100,000	27 (18.37)	78 (14.97)		25 (17.73)	76 (15.05)	
**BMI, mean (SD), kg/m2**	26.11 (4.60)	26.46 (5.42)	0.47	26.12 (4.54)	26.40 (5.44)	0.58
**Breathalyzer, n (%)**						
Above 0.05	0 (0)	0 (0)	1.00	0 (0)	0 (0)	1.00
Above 0.08	0 (0)	0 (0)		0 (0)	0 (0)	
**Age at First Alcohol Use, n (%)**						
< 14 years	26 (17.69)	27 (5.18)	***<0.001***	24 (17.02)	25 (4.95)	**<0.001**
15 – 16 years	51 (34.69)	97 (18.62)		48 (34.04)	93 (18.42)	
17 – 18 years	59 (40.14)	128 (24.57)		59 (41.84)	126 (24.95)	
19 – 20 years	5 (3.40)	78 (14.97)		5 (3.55)	75 (14.85)	
21+ years	6 (4.08)	151 (7.68)		5 (3.55)	147 (29.11)	
Missing	0 (0)	40 (7.68)		0 (0)	39 (7.72)	
**DSM Alcohol Symptoms, mean (SD)**	2.84 (0.98)	0 (0)	***<0.001***	2.82 (0.97)	0 (0)	**<0.001**
**Times Used Marijuana, n (%)**						
Never	27 (18.37)	326 (62.57)	***<0.001***	26 (18.44)	313 (61.98)	**<0.001**
≤10	59 (40.14)	132 (25.34)		57 (40.43)	130 (25.74)	
≥11 and ≤100	35 (23.81)	38 (7.29)		34 (24.11)	37 (7.33)	
>100 and ≤999	13 (8.64)	16 (3.07)		13 (9.22)	16 (3.17)	
≥1000	14 (8.64)	9 (1.73)		11 (7.80)	9 (1.78)	
**Times Used Illicit Drugs (other than Marijuana), n (%)**						
Never	93 (63.27)	467 (89.64)	***<0.001***	90 (63.83)	453 (89.70)	**<0.001**
≤10	35 (23.81)	44 (8.45)		34 (24.11)	43 (8.51)	
≥11 and ≤25	6 (4.08)	6 (1.15)		6 (4.26)	6 (1.19)	
>25	13 (8.84)	4 (0.77)		11 (7.80)	3 (0.59	
**FTND Score, mean (SD)**	2.00 (2.05)	1.91 (2.01)	0.87	1.88 (1.93)	1.88 (1.99)	0.99

Group differences were assessed with analyses of variance (continuous variables) and chi-square tests (categorical variables).

*AUD* alcohol use disorder, *BMI* body mass index, *DSM* diagnostic statistical manual, *FTND* fagerstrom test for nicotine dependence, *SD* standard deviation.

### MRI processing

The HCP neuroimaging data were acquired using a 3T Connectome Skyra scanner (Siemens). For detailed neuroimaging parameters and preprocessing, see the previously published data acquisition plan [35] and preprocessing conducted as part of the HCP minimally preprocessed dataset [36]. For each participant, two fMRI resting-state runs from each scan day (14 min 33 s, 1200 volumes each) were concatenated into a single 29 min resting-state scan for REST1 and a 29 min scan for REST2.

### Functional connectivity and graph metric calculation

The REST1 and REST2 scans were parcellated using Glasser’s multi-modal atlas (360 cortical parcels) [37] and 19 CIFTI subcortical parcels [36]. Each participant’s functional connectivity matrix (379×379 parcels) was computed using Fisher z-transformed Pearson’s correlation coefficients. The graph analysis focused on five commonly used metrics spanning different aspects of global and local functional topological organization: global functional integration (global efficiency), local functional integration/global segregation (local efficiency), modularity (participation coefficient), and centrality (betweenness centrality; degree/strength). For an overview and further details, see Supplementary Methods and Supplementary Fig. 2A.

### Identification of neuroimaging-based subtypes

We used HYDRA [32] semi-supervised clustering to evaluate the existence of neurobiological subtypes of AUD. Briefly, HYDRA works by iteratively classifying and clustering data based on pattern differences between a labelled ‘reference’ (e.g., controls) and ‘target’ group (e.g., patients). Unlike traditional unsupervised methods that cluster individuals by similarity, HYDRA clusters disease-related deviations, capturing pathological processes more precisely while reducing confounding variation (Supplementary Fig. 1) [26, 32].

HYDRA was applied using the five graph theory metrics calculated from REST1 as input, including age and sex/gender as covariates. We selected the optimal number of clusters (*k*) by evaluating cluster solutions from *k* = 2 to 10 using 10-fold cross-validation based on the Adjusted Rand Index (ARI) [38]. We ran each model 100 times, yielding 100 ARI observations for each combination of cluster solution (*k* = 2,…,10) and graph metric. We conducted a two-way repeated measures ANOVA (*car* package in R [v4.4.0, R Project for Statistical Computing]), with cluster (*k*) and graph theory metric as within-subject factors, to examine the performance across cluster solutions and metrics. Post-hoc analyses of significant main and interaction effects were performed (*emmeans* package R [v4.4.0, R Project for Statistical Computing]), applying a 5% false discovery rate (FDR) correction across all comparisons. The cluster solution and graph metric with the highest mean ARI across iterations, indicating the most stable solution, were selected for subsequent analyses (see Supplementary Fig. 2B and Supplementary Fig. 3). To further characterize the statistical properties of the selected graph metric, we evaluated between-subject variability and test-retest reliability for this metric across Control and AUD groups using data from both scan days (REST1 and REST2; see Supplementary Methods for details).

### Reproducibility analysis of AUD subtypes

Using the top-performing graph theory metric, we performed extensive reproducibility analyses to evaluate the robustness of the identified cluster solution (see Supplementary Methods and Supplementary Fig. 2C). First, we implemented resampling-based validation [39], generating multiple iterations of perturbed data to evaluate subtype stability in relation to sampling variability. Second, we conducted a permutation test [40] to assess statistical significance of the optimal *k* cluster solution. Statistical significance was set at 0.05 for a two-tailed test. Third, we evaluated the temporal stability [41] of the cluster solution to examine the consistency of subtype assignment and replicability of the neuroimaging signatures over time, using the REST2 scans. Lastly, we evaluated whether individual differences in global signal (GS) intensity may have influenced our results by testing for group differences in GS intensity and evaluating the impact of including GS intensity as a covariate in the HYDRA pipeline (see Supplementary Methods for details).

### Subtype resting-state graph theory characterization

Upon establishing stability, we subsequently characterized the distinct neuroimaging signatures of the derived subtypes **(**Supplementary Fig. 2D**)**. Group comparisons (i.e., biotype-control; between biotype) were performed using multiple linear regression models (MATLAB, R2023b, MathWorks). Age and sex/gender were included as fixed effects, with group as the variable of interest, and whole-brain resting-state graph theory metrics as response variables. P-values were corrected for multiple comparisons across 379 parcellated brain regions at *p-FDR* < 0.05.

### Traditional case-control comparison

As a control analysis to the subtyping, we conducted case-control comparisons between the categorically-defined AUD group (n = 147) and controls (n = 521) to assess whether this standard approach captures neurobiological variation comparable to the derived AUD subtypes **(**Supplementary Fig. 2D**)**. This analysis also served as a ‘baseline’ to address HYDRA’s limitation of not evaluating whether a *k* = 1 cluster solution is optimal.

### Subtype phenotypic and clinical characterization

Demographics, phenotypic and clinical data, not included for clustering with HYDRA, were used as independent clinical validators of the derived subtypes (see Supplementary Methods and Supplementary Fig. 2D). Complete lists of included phenotypic and clinical data are provided in Supplementary File 1: Table 1**and** Supplementary File 1: Table 2. To reduce dimensionality, all 100 phenotypic/clinical measures were submitted to an Exploratory Factor Analysis, as described in [42] (see Supplementary Methods for details). Between-group comparisons were examined using a series of multivariate linear mixed models (*lme4* package, R [v4.4.0, R Project for Statistical Computing]). Models included a fixed effect of group (cluster, controls), variable (measures included), group-by-variable interaction, and covariates (age, sex/gender). To account for sibling status, Family-ID was included as a random intercept, uniquely identifying each biological family. Post-hoc analyses examining significant fixed effects were performed (*emmeans* package, R [v4.4.0, R Project for Statistical Computing]), applying a 5% FDR correction across all comparisons.

We additionally assessed predictive validity by conducting multinomial logistic regression analyses (*nnet* package, R [v4.4.0, R Project for Statistical Computing]). Group membership (Controls, Subtype 1, Subtype 2) was the outcome and EFA-derived phenotypic factors were the predictors, adjusting for age and sex/gender. Models were first estimated with Controls as the reference group and then re-estimated with Subtype 1 as the reference group to directly compare subtypes. Significance was determined using Wald tests with Benjamin-Hochberg FDR correction applied across phenotypic factors and contrasts. Effect sizes were reported as odds ratios (ORs).

### Subtype moderation of brain-behavior and phenotype-AUD severity associations

We then evaluated whether brain-behavior associations differed between groups. To reduce the multiple comparisons burden, we computed the mean participation coefficient for each of the Glasser 22 networks originally used for participation coefficient derivation and for each subject. We then fitted regression models for each of the EFA-derived phenotypic factor (‘Phenotype’) that included the mean network participation coefficient, Group (AUD vs Controls; HYDRA Subtypes vs Controls), and their interaction (Participation Coefficient x Group), adjusting for age and sex/gender. P-values were FDR corrected across phenotypes.

To additionally test whether the subtype membership moderated associations between phenotypic factors and alcohol symptom severity differentially, we conducted linear regression analyses within the subtypes, using DSM AUD symptom count as a continuous index of severity. For each EFA-derived phenotypic factor, we fit models that included phenotype, subtype, and their interaction (Phenotype x Subtype) as predictors of symptom severity, adjusting for age and sex/gender. Interaction terms were evaluated using FDR correction (p < 0.05).

### Generalizability analysis including comorbid AUD and cannabis dependence

Finally, we evaluated whether including individuals with comorbid AUD and cannabis dependence generated a subtype solution that generalized to or meaningfully differed from our original subtype solution derived from AUD individuals without cannabis dependence (‘AUD-ONLY’). To test this, we reran the entire HYDRA pipeline, including subtype identification and reproducibility analyses (see Supplementary Methods for details**)**, using the initial AUD-ONLY sample (N = 147) and adding individuals with comorbid AUD and DSM-IV cannabis dependence (‘AUD-CD’: N = 55). This yielded a total ‘combined’ AUD/AUD-CD sample of N = 202. This new combined AUD/AUD-CD sample had significantly lower education levels and higher use of cannabis and other illicit drugs than the AUD-ONLY sample (Supplementary File 2: Table 1**)**. Importantly, the AUD-ONLY and AUD/AUD-CD samples did not differ in alcohol or tobacco use characteristics (Supplementary File 2: Table 2).

## Results

### Two neurobiological subtypes revealed by HYDRA

**Fig. 1:**
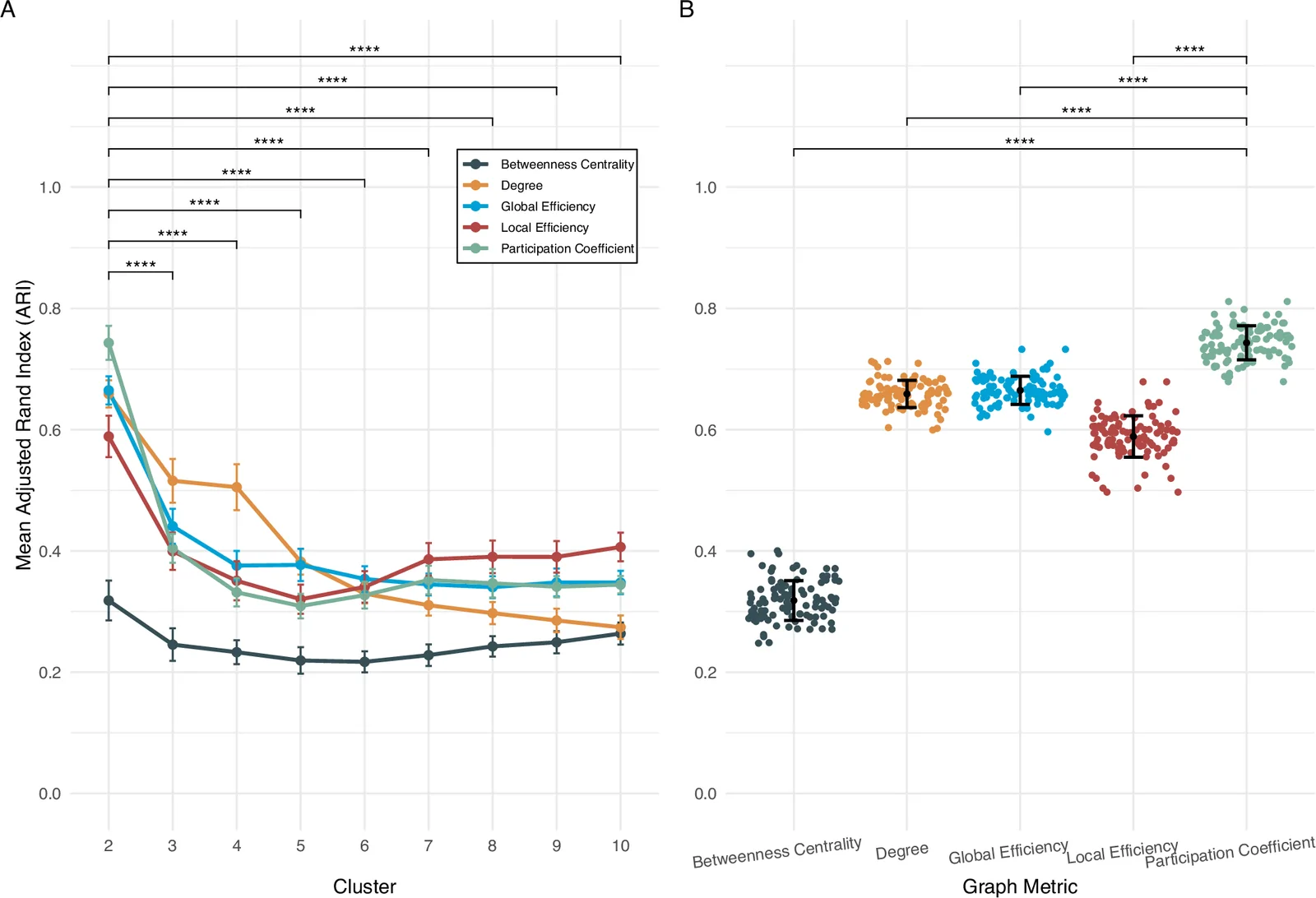
Cluster and Graph Metric Selection for HYDRA Subtyping. Cluster solutions (*k* = 2,…,10) were evaluated using 10-fold cross-validation based on the Adjusted Rand Index (ARI) across 100 iterations of HYDRA, per *k* and graph metric. A two-way repeated measures ANOVA assessed performance across cluster solutions and metrics. **A** The cluster solution (*k* = 2) and (**B**). graph metric (participation coefficient) with the highest average ARI were selected for all subsequent analyses. **** *p-FDR* < 0.0001.

A significant interaction between Cluster (*k*) and graph theory metric was identified (*F*(32) = 587.22, *p-FDR* < 0.001, ηp² = 0.81; Supplementary File 1: Table 3). Post-hoc comparisons indicated that, across all repeated iterations, the 2-cluster solution (*k* = 2; mean [SD] ARI, 0.59 [0.15]) consistently outperformed all other cluster solutions for each graph theory metric (*p-FDR* < 0.001; Fig. 1A). Among the five different graph metrics, the participation coefficient demonstrated the highest reliability (mean [SD] ARI, 0.74 [0.03]), significantly greater than all other metrics (*p-FDR* < 0.001, Fig. 1B). Results were consistent across thresholded and weighted graph theory metrics (Supplementary Fig. 4). Further, the participation coefficient exhibited comparable between-subject variability and test-retest reliability in Controls and AUD, indicating that the HYDRA subtype solution is unlikely to be driven by systematic differences in measurement variance or reliability between groups (Supplementary Results**;** Supplementary Fig. 5).

**Fig. 2:**
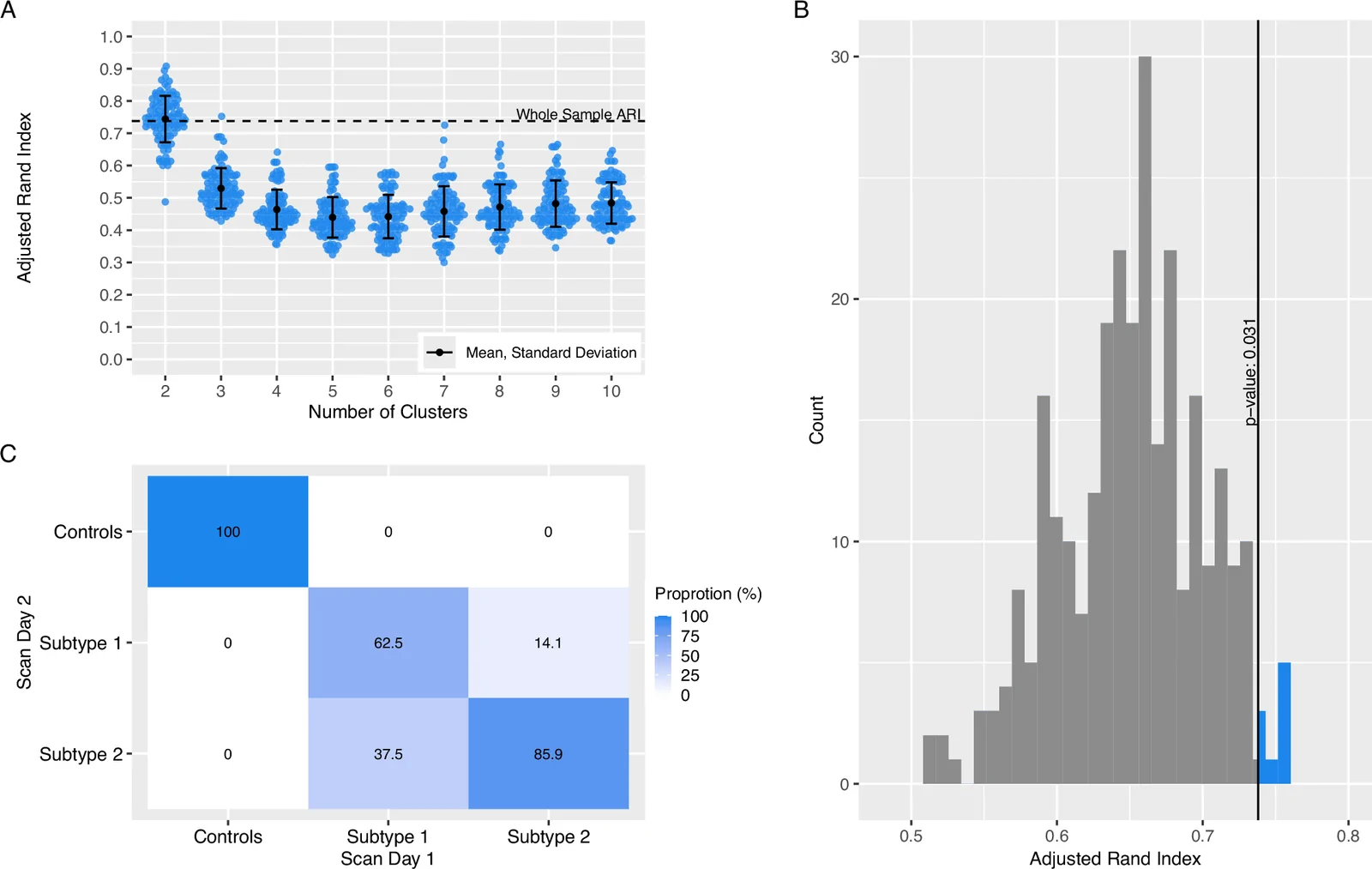
Validation of HYDRA-Derived Subtypes Using Participation Coefficient. **A** Resampling-Based Validation: Adjusted Rand Index (ARI) for clustering solutions (*k* = 2,…,10), computed across 100 HYDRA iterations using 80% stratified subsamples with replacement. **B** Permutation Test: ARI stability across 285 permuted samples (Cohen’s d = 2.00; 95% CI, 0.08–0.10; *p* < 0.05). **C** Temporal Stability Analysis: Stability of subtype assignment across scan days.

**Fig. 3:**
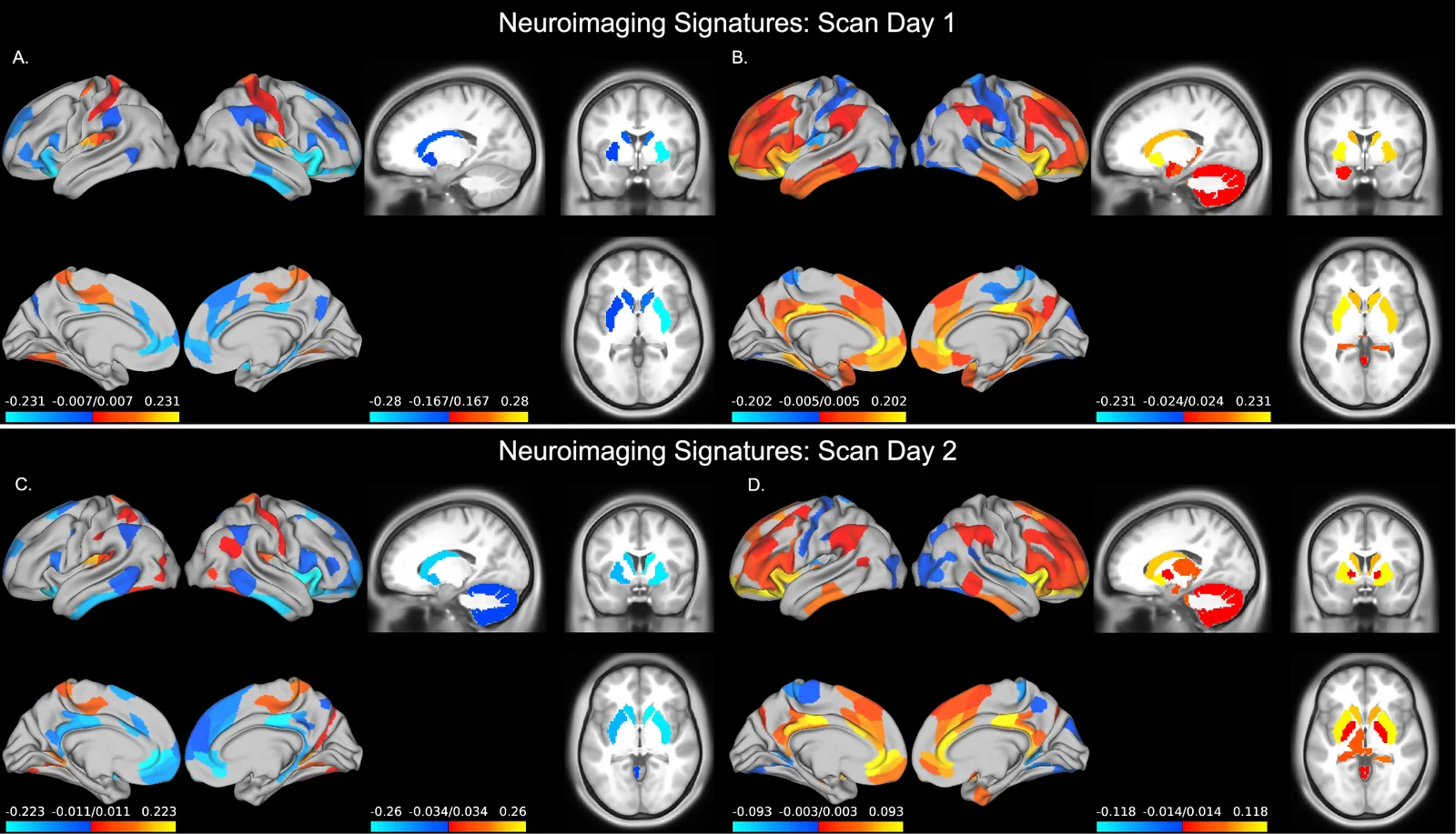
Neuroimaging Signatures of HYDRA-Derived Subtypes. Subtype-specific alterations in participation coefficient relative to controls. A–B Neuroimaging signatures from Scan Day 1 (REST1): **A.** Subtype 1. **B.** Subtype 2. C–D Replication of subtype-specific patterns in Scan Day 2 (REST2): **C.** Subtype 1. **D.** Subtype 2. All regions shown are significant at *p-FDR* < 0.05. Color bar represents standardized beta weights.

The optimal cluster solution (*k* = 2), using the most reliable graph theory metric (participation coefficient), was further validated through resampling-based validation, permutation testing, and temporal stability analysis. In resampling-based validation, 99 of 100 iterations identified *k* = 2 as the optimal solution (mean [SD] ARI, 0.74 [0.07]; Fig. 2A). The ARI of the 2-cluster solution was significantly higher than the null distribution in the permutation test (Cohen’s *d* = 2.00; 95% CI, 0.08–0.10; *p* < 0.05; Fig. 2B). Lastly, temporal stability analysis demonstrated high model similarity between REST1 and REST2 solutions (ARI = 0.96), with generally consistent subtype-assignment (Subtype 1: 35 of 47 [63%]; Subtype 2: 73 of 85 [86%]; Fig. 2C), and robust replication of neuroimaging signatures across acquisition days (Fig. 3A-D). Therefore, we present the results of the participation coefficient-derived 2-cluster solution for all subsequent analyses.

### Neuroimaging signatures of HYDRA-Derived subtypes

HYDRA Subtype 1 demonstrated greater participation coefficient in the ‘sensory-motor network’, encompassing regions of the somatosensory, sensorimotor, parietal association, and visual networks, but lower participation coefficient in the ‘association network’, comprising regions of the frontoparietal, default-mode, reward, salience, and subcortex, including bilateral putamen and caudate (*p-FDR* < 0.05, Figs. 3A, C, Supplementary File 1: Table 4). HYDRA Subtype 2 exhibited an inverse pattern, characterized by greater participation coefficient in the association network, including regions of the frontoparietal, default-mode, reward, salience and limbic networks, and subcortex - bilateral caudate, putamen, cerebellum, hippocampus, and right amygdala - with lower participation coefficient across the sensory-motor network (*p-FDR* < 0.05, Figs. 3B, D, Supplementary File 1: Table 5). Results remained highly consistent after additionally controlling for global signal standard deviation (GS SD), indicating that global signal variability did not account for the observed effects (Supplementary Methods and Results; Supplementary Fig. 6, Supplementary Fig. 7; Supplementary File 1: Table 6**)**. Subtype-control comparisons across all other calculated graph theory metrics supported the inverse pattern of the participation coefficient-derived subtypes (Supplementary File 1: Table 7, 8); between-subtype results are provided in Supplementary File 1: Table 9.

#### Traditional case-control comparison

Following correction (*p-FDR* < 0.05), case-control comparisons (categorically-defined AUD vs controls) revealed a significant increase in betweenness centrality in only 1 of 379 parcels - the left frontal eye field – with no other significant differences across all other graph metrics (Supplementary File 1: Table 10**)**.

### Subtype phenotypic and clinical characterization

**Fig. 4:**
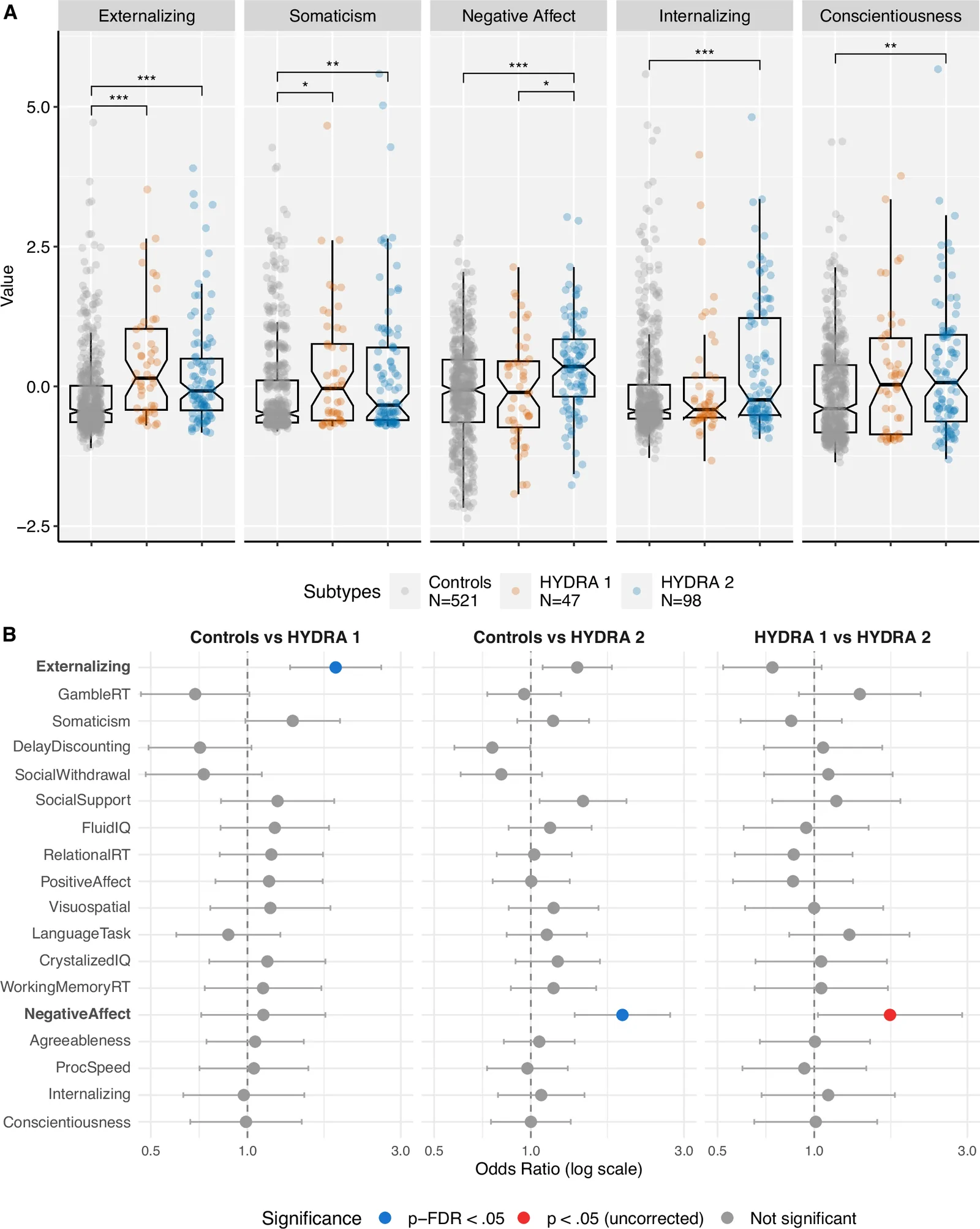
Phenotypic Characterization and Clinical Predictive Validity of Derived Subtypes. **A** Extracted 18 phenotypic/clinical latent factors via Exploratory Factor Analysis from all the HCP measures (100 variables) and compared groups. Only factors demonstrating significant group differences are displayed. See Supplementary **File 1:** Table 12 for all comparisons on the derived latent factors. *p-FDR* * <0.05; ** <0.01; ***<0.001. **B** Results from multinomial logistic regression testing predictive validity of subtype membership using EFA-derived phenotypic factors as predictors. Odds ratios reflect effects per one standard deviation increase in the predictor. Blue dot: *p-FDR* < 0.05. Red dot: *p-uncorrected* <0.05.

The Exploratory Factor Analysis identified 18 latent factors underlying the phenotypic/clinical variable space **(see Supplementary Results**, Supplementary Fig. 8**, and** Supplementary File 1: Table 11). HYDRA subtype 1 was characterized by the highest levels of externalizing behaviors (Cohen’s *d* = −0.63, 95% CI [−0.89, −0.23], *p-FDR* < 0.001) and somaticism (Cohen’s *d* = −0.41, 95% CI [−0.70, −0.03], *p-FDR* < 0.05; (Fig. 4A, Supplementary File 1: Table 12). HYDRA subtype 2 demonstrated the highest levels of negative affect (Cohen’s *d* = −0.58, 95% CI [−0.77, −0.28], *p-FDR* < 0.001) and internalizing (Cohen’s *d* = −0.48, 95% CI [−0.68, −0.19], *p-FDR* < 0.001), as well as high conscientiousness/attention (Cohen’s *d* = −0.39; 95% CI [−0.60, −0.11], *p-FDR* < 0.01), and elevated somaticism (Cohen’s *d* = −0.39, 95% CI [−0.60, −0.11, *p-FDR* < 0.01), and externalizing behaviors (Cohen’s *d* = −0.41, 95% CI [−0.61, −0.12], *p-FDR* < 0.001; (Fig. 4A, Supplementary File 1: Table 12). Between the subtypes, negative affect was the only factor that significantly differentiated them (Cohen’s *d* = −0.43, 95% CI [−0.77, −0.01], *p-FDR* < 0.05; Fig. 4A**;** Supplementary File 1: Table 12).

The covariate-adjusted multinomial logistic regression models indicated that greater externalizing symptoms were associated with increased odds of HYDRA Subtype 1 membership relative to Controls (OR = 1.88, 95% CI [1.36, 2.61], *p-FDR* < 0.01; Fig. 4B**)**. In contrast, higher negative affect was associated with increased odds of HYDRA Subtype 2 membership relative to Controls (OR = 1.93, 95% CI [1.37, 2.72], *p-FDR* < 0.01; Fig. 4B). In direct comparisons between the two HYDRA subtypes, negative affect differentiated the groups, such that higher negative affect was associated with greater odds of HYDRA Subtype 2 membership relative to Subtype 1 (OR = 1.72, 95% CI [1.03, 2.89], *p* = 0.04, uncorrected; Fig. 4B).

No significant differences were demonstrated between the subtypes across demographics (Table 2), alcohol and drug use characteristics (Supplementary File 1: Table 13), or their distribution of individuals with mild versus moderate-severe AUD (Table 2). Regarding alcohol use characteristics, as expected, both subtypes demonstrated greater levels of alcohol use as compared to controls (*p-FDR* < 0.05*;* Supplementary File 1: Table 13).

**Table 2:** HYDRA Subtype Demographics.

Characteristic	HYDRA Subtype 1 (n = 49)	HYDRA Subtype 2 (n = 98)	Significance (*p-value*)
**Age, mean (SD), years**	29.29 (3.49)	28.36 (3.15)	0.11
**Sex/gender,** ***n*** **(%)**			
Male	33 (67.35)	55 (56.12)	0.26
Female	16 (32.65)	43 (43.88)	
**Race,** ***n*** **(%)**			
Asian	4 (8.16)	4 (4.08)	0.83
Black/AA	3 (6.12)	9 (9.18)	
Caucasian	39 (79.59)	78 (79.59)	
First Nations	0 (0)	1 (0.19)	
Multiracial	2 (4.08)	4 (4.08)	
Unknown	1 (2.04)	3 (3.06)	
**Education, mean (SD), years**	15.41 (1.44)	15.26 (1.56)	0.57
**Income, $**			
< 50,000	24 (48.98)	49 (50.00)	0.99
≤50,000 and <100,000	16 (32.65)	31 (31.63)	
≥100,000	9 (18.37)	18 (18.37)	
**BMI, mean (SD), kg/m**^**2**^	26.88 (4.66)	25.74 (4.54)	0.16
**Age at First Alcohol Use, n (%)**			
< 14 years	12 (24.49)	14 (14.29)	0.38
15 – 16 years	13 (26.53)	38 (38.78)	
17 – 18 years	19 (38.78)	40 (40.82)	
19 – 20 years	2 (4.08)	3 (3.06)	
21+ years	3 (6.12)	3 (3.06)	
**DSM Alcohol Symptoms, mean (SD)**	2.96 (1.06)	2.72 (0.91)	0.95
**DSM AUD Severity, n (%)**			
**Mild (2-3 symptoms)**	33 (67.34)	76 (77.55)	0.26
**Moderate to severe (4-5+ symptoms)**	16 (32.65)	22 (22.45)	
**Times Used Marijuana, n (%)**			
Never	7 (14.29)	20 (20.41)	0.91
≤10	21 (42.86)	38 (38.78)	
≥11 and ≤100	12 (23.81)	23 (23.47)	
>100 and ≤999	5 (10.20)	8 (8.16)	
≥1000	4 (8.16)	9 (9.18)	
**Times Used Illicit Drugs (other than Marijuana), n (%)**			
Never	31 (63.27)	62 (63.27)	0.72
≤10	10 (20.41)	25 (25.51)	
≥11 and ≤25	2 (4.08)	4 (4.08)	
>25	6 (12.24)	7 (7.14)	
**FTND Score, mean (SD)**	1.87 (1.84)	2.10 (2.21)	0.69

Group differences were assessed with analyses of variance (continuous variables) and chi-square tests (categorical variables).

*AUD* alcohol use disorder, *BMI* body mass index, *DSM* diagnostic statistical manual, *FTND* fagerstrom test for nicotine dependence, *SD* standard deviation.

### Moderation of brain-behavior and phenotype-AUD severity associations

Brain-behavior moderation analyses revealed no significant interaction effects across any of the 18 latent factors after FDR correction (Supplementary File 1: Table 14), indicating that brain-behavior associations were similar between both AUD group versus Controls, and the HYDRA-derived Subtypes versus Controls. Moderation analysis examining the association between phenotypic latent factors and DSM AUD symptom severity did not uncover any significant interaction after FDR correction (Supplementary File 1: Table 15). This suggests that the relationship between phenotype and symptom severity was broadly similar across HYDRA-derived subtypes.

### Subtype solution generalizability when including AUD with comorbid cannabis dependence

The HYDRA-derived subtype solution in the combined AUD/AUD-CD sample uncovered subtypes that were highly consistent with those derived from the AUD-ONLY sample. As in the original solution, *k* = 2 subtypes were identified (ARI = 0.75) that were robust to resampling-based validation and permutation testing (*p-FDR* < 0.05; Supplementary Fig. 9A, B). The overlap in the cluster assignments between the initial AUD-ONLY solution and the new combined AUD/AUD-CD solution was extremely high (ARI = 0.99; Supplementary Fig. 9C), indicating that inclusion of individuals with comorbid AUD-CD did not meaningfully alter the AUD cluster solutions. Accordingly, the neuroimaging signatures of the newly derived AUD/AUD-CD subtypes closely replicated those in the original analysis (Supplementary Fig. 10). Specifically, consistent with the original solution, Subtype 1 demonstrated greater participation coefficient in the ‘sensory motor network’ and lower participation coefficient in the ‘association networks’, while Subtype 2 demonstrated the inverse pattern: greater participation coefficient in the association and lower in the sensory-motor networks.

Moreover, the AUD/AUD-CD HYDRA subtypes exhibited phenotypic profiles that were consistent with those identified in the AUD-ONLY analysis (Supplementary Fig. 11). AUD/AUD-CD Subtype 1 retained a predominantly externalizing phenotypic profile, exhibiting the highest levels of externalizing and somaticism, consistent with the AUD-ONLY analysis. Similarly, AUD/AUD-CD Subtype 2 was characterized by the highest levels of negative affect and internalizing, also consistent with the AUD-ONLY analysis. In addition to these effects, the higher levels of illicit drug use (other than cannabis) in the new combined sample differentially affected the two new subtypes. Specifically, AUD/AUD-CD Subtype 1 exhibited significantly higher levels of illicit substance use than AUD/AUD-CD Subtype 2 (Supplementary File 2: Table 3), consistent with its predominantly externalizing phenotypic profile. There were no significant differences between the newly derived AUD/AUD-CD subtypes in regard to alcohol or tobacco use characteristics (Supplementary File 2: Table 4).

## Discussion

In the present study, we leveraged a combination of semi-supervised clustering and whole-brain resting-state graph theory analysis to evaluate the neurobiological heterogeneity of AUD. As hypothesized, we identified robust AUD subtypes with distinct, inverse neurobiological signatures. HYDRA Subtype 1 demonstrated greater participation coefficient in the sensory-motor network, but lower participation coefficient in the association network, while HYDRA Subtype 2 exhibited the inverse pattern, characterized by greater participation coefficient in the association network and lower participation coefficient across the sensory-motor network. Extensive validation confirmed the reproducibility of these two subtypes, and that they were further differentiated by unique phenotypic and clinical profiles. Specifically, Subtype 1 was characterized by the highest levels of externalizing behaviors and somaticism, while Subtype 2 demonstrated the highest levels of negative affect and internalizing symptoms, in combination with elevated somaticism, and externalizing behaviors. Consistent with these phenotypic profiles, the HYDRA-derived subtypes also demonstrated predictive validity: higher levels of externalizing significantly predicted greater odds of Subtype 1 membership relative to Controls, whereas higher negative affect predicted greater odds of Subtype 2 membership. Importantly, the two derived subtypes demonstrated comparable AUD symptom severity and a similar distribution of individuals with mild versus moderate-severe AUD, confirming that their distinct profiles were driven by mechanistic differences rather than differences in AUD severity. Additionally, including individuals with comorbid AUD and cannabis dependence did not meaningfully alter the subtype solution, neurobiological signatures or phenotypic and clinical profiles, supporting the robustness of the identified subtypes. Further, traditional case-control analyses failed to reveal meaningful differences, underscoring the need for approaches that uncover neurobiological variation. Together, these findings provide interpretable, replicable evidence of neurobiological heterogeneity in AUD, with direct clinical potential.

Our rigorous statistical procedure revealed two robust AUD subtypes, each defined by distinct abnormalities in whole-brain function. These patterns were best captured using the participation coefficient [43], a graph metric that quantifies ‘hubs’ [44] - brain regions (or “nodes”) that are integral for balancing integrative and segregative processing to support cost-efficient functioning [45]. Dysregulation in this balance – common across psychopathology [45, 46] including in AUD and other addictions [22, 47–49] – reflects disorganized brain network topology that impairs efficient information transfer and is linked to profound impairments in behavior [22, 48]. Furthermore, recent work in AUD supports the clinical utility of the participation coefficient, showing treatment-related attenuation of abnormalities in participation coefficient within two weeks of psychotherapy alone or combined psychotherapy-pharmacotherapy [49].

Our HYDRA-derived Subtype 1 was characterized by greater participation coefficient in the sensory-motor network and lower participation coefficient in the association network, including the bilateral caudate and putamen. The sensory-motor network is critical for integrating sensory information [50, 51] and modulating habitual behavior [50, 52]. It is considered a ‘transdiagnostic hub’ for impulsivity, central not only to addiction but also to other externalizing disorders like attention-deficit hyperactive disorder [53]. Dysregulation in both sensory-motor and association networks has been linked to antisocial behaviors, rule-breaking, poor planning and impulsive decision-making, all of which contribute to excessive substance use [51, 54]. Overall, Subtype 1’s distinct neuroimaging signature suggests an enhanced capacity for bottom-up sensory-motor engagement, potentially at the expense of top-down, higher-order processing and linked to the impulsive tendencies characteristic of externalizing behaviors. Consistent with this, Subtype 1 was characterized by the highest levels of externalizing behaviors and somaticism, and higher externalizing significantly predicted greater odds of Subtype 1 membership relative to Controls, further linking its neurobiological signature with disinhibition and behavioral dysregulation. Greater sensory-motor integration has also been demonstrated in independent AUD samples [47, 48], and its functional connectivity is highly predictive of AUD severity [55]. A recent meta-analysis also showed that sensory-motor region activation is convergent across substance use disorders, and strongly associated with craving, habitual action execution, and illness duration [56]. Moreover, the reduced participation coefficient in Subtype 1’s association network mirrors the neurobiological signature of the phenotypically derived ‘Reward’ subtype previously reported in AUD, which was characterized by disinhibited, approach-related behaviors, like social risk-taking and sensation-seeking [14]. This phenotypically derived AUD ‘Reward’ subtype is assumed to use alcohol primarily for positive reinforcement or ‘reward seeking’, as previously proposed [15]. Accordingly, it is primarily characterized by approach- and reward-related behaviors consistent with the Research Domain Criteria (RDoC) ‘positive valence’ domain [14, 57]. Taken together, the neurobiological signature of Subtype 1 reported here, may thus reflect a pathway to AUD that is highly associated with impulsive and reward-seeking behaviors, supporting the theorized ‘externalizing pathway’ to AUD [58, 59].

Conversely, HYDRA-derived Subtype 2 was characterized by greater participation coefficient in the association network and subcortex—including frontoparietal, default-mode, reward, salience and limbic networks and the caudate, putamen, amygdala, hippocampus, cerebellum and brain stem—along with lower participation coefficient in the sensory-motor network. This distinct pattern suggests an over-engagement of top-down, higher-order processes and subcortical functioning, which may override effective functioning. Indeed, aberrant resting-state functional connectivity in executive control, salience, and default-mode networks is consistently observed in AUD, suggesting an imbalance of top-down control, salience reactivity, and self-referential processing [18, 20, 60]. Particularly distinctive of this subtype is the hyper-engagement of the amygdala, hippocampus, and default-mode network, encompassing regions critical for emotion, affective salience, memory, and self-regulatory/referential processing [19, 61, 62]. This may reflect the hypersensitivity to emotional cues and difficulty disengaging from negative affective states characteristic of internalizing psychiatric conditions [18, 63]. Consistent with this interpretation, Subtype 2 showed a predominantly internalizing phenotypic and clinical profile, characterized by the highest levels of negative affect and internalizing symptoms, in addition to elevated somaticism and externalizing behaviors. In line with this phenotypic profile, higher negative affect predicted greater odds of Subtype 2 membership relative to Controls, supporting its clinical relevance and predictive validity. Overall, Subtype 2 thus demonstrated a complex profile of cognitive and emotional dysregulation, where impulsive behaviors may be driven by heightened negative affect. Indeed, substantial research supports the role of negative emotionality in driving substance use disorders [14, 17, 63–66]. Notably, emotion dysregulation, like behavioral disinhibition, stems from an imbalance between top-down prefrontal control and bottom-up subcortical systems, but distinctively involves limbic circuitry, such as the amygdala [59]. Our findings underscore this crucial distinction: compared to Subtype 1’s neuroimaging signature and associated externalizing phenotype, Subtype 2 uniquely engaged subcortical limbic structures. Overall, Subtype 2 exhibited a neurobiological signature linked to both internalizing and externalizing behaviors, distinguishing it from the phenotypically-derived ‘Relief’ type previously reported in AUD [14, 15, 67]. This previously identified ‘Relief’ subtype is characterized by a purely internalizing profile driven by negative affect and internalizing psychopathology [14, 15, 67], and marked exclusively by behaviors within the RDoC ‘negative valence’ domain [14, 57], in contrast to the more complex profile observed in Subtype 2. These differences may stem from the higher dimensionality and sensitivity of neuroimaging data relative to phenotypic assessments. Thus, the complex profile of Subtype 2 identified in this study underscores the value of neuroimaging-based subtyping in recovering clinically meaningful subtypes, showcasing its ability to capture intricate patterns beyond those captured by traditional phenotypic subtyping approaches.

Additional analyses demonstrated that brain-behavior associations were similar between Controls and the HYDRA-derived Subtypes. This suggests that the structure of brain-behavior associations is shared rather than qualitatively reorganized in AUD. At the same time, significant mean-level phenotypic differences across HYDRA subtypes, along with logistic regression results showing that specific phenotypic factors distinctly predicted subtype membership, indicates that individuals with AUD primarily differ in their positions along these shared brain-behavior dimensions. We further did not find evidence that subtype membership moderated the association between phenotypes and AUD symptom severity. This suggests that the relationship between behavioral dimensions and clinical severity scales was also similar across subtypes. This interpretation is consistent with our findings that subtypes did not significantly differ in DSM AUD symptoms, severity, or other clinical use variables.

Providing further evidence of robustness and generalizability, the HYDRA-derived subtype solution remained highly stable following the inclusion of individuals with comorbid AUD and cannabis dependence into the AUD sample. The persistence of the robust two-subtype structure, the near perfect overlap between cluster assignments, and highly replicable neuroimaging signatures indicate that the identified subtype solution is not contingent on a narrowly defined sample of AUD individuals without comorbid cannabis dependence. Regarding their phenotypic characterization, the core profiles were largely preserved: Subtype 1 continued to exhibit a predominantly externalizing profile, and Subtype 2 a complex profile driven primarily by internalizing. Finally, the inclusion of individuals with comorbid AUD and cannabis dependence slightly altered the substance use characteristics of Subtype 1, such that Subtype 1 demonstrated significantly higher levels of illicit drug use other than cannabis, consistent with its externalizing profile. Taken together, the foundational neurobiological signatures and phenotypic characterization of the subtypes in the initial AUD-ONLY sample were largely preserved in this additional analysis. Given the high prevalence of comorbid cannabis use disorder in individuals with AUD, these results strengthen the generalizability and translational relevance of our findings.

The need for personalized medicine approaches is now widely acknowledged as essential to improving treatment outcomes for AUD [9, 11, 15, 67], as well as across addiction [6, 10, 14, 68] and psychiatry more broadly [8, 16, 28, 69–72]. However, progress has been constrained by a currently limited mechanistic understanding and significant barriers with the practical implementation of personalized medicine approaches in clinical care [69, 73–75]. As a result, “one-size-fits-all” treatments remain the standard for AUD, despite inconsistent outcomes given the vast heterogeneity underlying the disorder [10, 11, 22]. Our results provide an opportunity for more targeted allocation of existing treatments and the development of subtype-specific interventions that directly address the distinct and malleable [22] profiles of brain network dysregulation and their associated characteristics. Emerging treatments like neuromodulation, effective in depression [29, 30], hold promise for directly attenuating brain network dysregulation. However, while initial results are promising [76, 77], neuromodulation trials in substance use disorders remain inconclusive, with no consensus on stimulation targets or protocols (excitatory vs inhibitory stimulation) [22, 78, 79]. Our subtype-specific findings may provide the clarity needed to guide and refine these neuromodulation approaches, as well as inform broader treatments. For instance, given the neurobiological profile of Subtype 1, excitatory non-invasive brain stimulation targeting the more segregated nodes of the executive control and salience network may potentially provide targeted benefits reducing impulsivity and cue-induced craving [52, 78]. Interventions addressing this subtype’s associated externalizing phenotype, such as behavioral strategies targeting disinhibition or approach-related behaviors and motivations [80, 81], or medications like naltrexone, demonstrating more efficacy in those who drink for ‘reward’ rather than ‘relief’ motivations [15, 67], may also be particularly effective. For Subtype 2, stimulation protocols aimed at attenuating the hyperconnectivity of emotion and executive-control circuits may be more beneficial [78, 79]. This subtype may also respond well to treatments focused on affect regulation and or enhancing tolerance to negative emotions, such as pharmacotherapies for anxiety/depression effective in those with comorbid alcohol dependence [82] or specialized cognitive-behavioral therapy for substance use disorders with comorbid mental health disorders [83]. By aligning interventions with distinct neurobiological profiles and their associated phenotype, these results offer a powerful framework for more effective, individualized AUD treatment.

This study, to our knowledge, presents the first application of neuroimaging-based psychiatric subtyping in AUD, with only one previous study examining neurobiological heterogeneity in individuals reporting any level of alcohol misuse [31]. Previous efforts have primarily relied on standard unsupervised clustering approaches [8, 26], which are susceptible to non-pathological confounds [8, 26], and using (complex) feature reduction techniques to handle high-dimensional neuroimaging data [8, 26, 31] compromising the interpretability and clinical applicability of the resulting subtypes [8, 26, 31]. The sole previous investigation of alcohol misuse biotypes illustrate these challenges: its subtypes were derived from complex resting-state feature transformations, which, as noted by the authors, ultimately prevented the interpretation of subtype-specific features distinguishing each type [31], limiting their potential for direct clinical translation. To address these limitations, we combined a semi-supervised approach, designed to cluster pathologically relevant variations more effectively [26, 32], with whole-brain graph theory metrics as input. This approach avoids complex feature reduction methods, instead deriving subtypes based on whole-brain topology and processing [22]. As a result, distinct, interpretable and subtype-specific neurobiological signatures were identified, using a graph metric shown to attenuate with treatment in AUD [49]. Extensive validation further supported the reproducibility and clinical relevance of our findings, underscoring the potential of our approach for advancing biomarker detection and personalized treatments for AUD.

While this study uses a statistically rigorous approach, there are limitations. Essential to our aim of identifying robust neuroimaging-based subtypes, the HCP dataset provided a multimodal sample including nearly 60 min of rs-fMRI data per subject across two scan days. These scan parameters exceed recommendations (i.e., ≥ 30 min per subject) for optimizing reliable rs-fMRI estimates [84–88], and enabled us to assess the temporal stability of our subtypes, an added layer of validation essential for evaluating their utility [8, 41]. However, as a population-based sample not primarily focused on alcohol use, the majority of the AUD sample in the HCP included individuals with mild AUD, who were likely non-treatment seeking. While improving treatment efforts across all levels of AUD severity is a major public health goal, especially to enable early intervention and mitigate the progression to more severe stages and their detrimental consequences [89–93], future research evaluating the generalizability of these subtypes to a more severe or treatment-seeking AUD population is warranted. Moreover, while the dataset included deeply phenotyped information that supported subtype clinical interpretation, it lacked more fine-grained measures of alcohol use and clinical assessments, such as those on treatment history, outcomes, or withdrawal, that could have provided additional insight for subtype characterization. Furthermore, the cross-sectional design limits our ability to determine whether the identified subtypes predate the onset of AUD or arise as a consequence of the disorder. Longitudinal studies are needed to examine subtype-specific changes and outcomes over time. Another important consideration are sex/gender differences [94]. As in prior studies using HYDRA [95–98], we covaried for sex/gender in our subtyping analysis. Future research using a sufficiently large, sex/gender-balanced sample should investigate whether subtypes stratified by sex/gender emerge.

## Conclusion

This study is the first to characterize neurobiological heterogeneity in AUD, revealing, as hypothesized, distinct subtypes with unique, complex and interpretable neurobiological profiles, and demonstrable clinical relevance and predictive validity. Findings underscore the value and direct clinical potential of brain-based subtyping to guide the development of personalized and neurobiologically-informed targeted interventions for AUD.

## Supplementary information

Supplementary Methods, Results, Figures

Supplementary Tables File 1

Supplementary Tables File 2

## Data Availability

Data used in this study were provided by the Human Connectome Project, WU-Minn Consortium (1U54MH091657) funded by the NIH Blueprint for Neuroscience Research. These data are publicly available following completion of a data use agreement (https://www.humanconnectome.org/study/hcp-young-adult/document/1200-subjects-data-release).

## Code availability

HYDRA code used in this study is available at https://github.com/evarol/HYDRA.

## References

[1] Substance Abuse and Mental Health Services Administration. Key Substance Use and Mental Health Indicators in the United States: Results from the 2023 National Survey on Drug Use and Health. Center for Behavioral Health Statistics and Quality, Substance Abuse and Mental Health Services Administration; 2024. (NSDUH Series H-59). Report HHS Publication No. PEP24-07-021. https://www.samhsa.gov/data/report/2023-nsduh-annual-national-report.

[2] Esser MB. Deaths from excessive alcohol use — United States, 2016–2021. MMWR Morb Mortal Wkly Rep. 2024;73:154–61. 10.15585/mmwr.mm7308a1 PMC10907037 38421934

[3] Organization WH. Global Status Report on Alcohol and Health 2018. World Health Organization; 2019.p.472.

[4] Mekonen T, Chan GCK, Connor J, Hall W, Hides L, Leung J. Treatment rates for alcohol use disorders: a systematic review and meta-analysis. Addiction. 2021;116:2617–34. 10.1111/add.15357 33245581

[5] Sliedrecht W, de Waart R, Witkiewitz K, Roozen HG. Alcohol use disorder relapse factors: a systematic review. Psychiatry Res. 2019;278:97–115. 10.1016/j.psychres.2019.05.038 31174033

[6] Pettorruso M, Di Lorenzo G, De Risio L, Di Carlo F, d’Andrea G, Martinotti G. Addiction biotypes: a paradigm shift for future treatment strategies? Mol Psychiatry. 2024;29:1450–2. 10.1038/s41380-024-02423-w 38243073

[7] Feczko E, Miranda-Dominguez O, Marr M, Graham AM, Nigg JT, Fair DA. The heterogeneity problem: approaches to identify psychiatric subtypes. Trends Cognit Sci. 2019;23:584–601. 10.1016/j.tics.2019.03.009 PMC6821457 31153774

[8] Brucar LR, Feczko E, Fair DA, Zilverstand A. Current approaches in computational psychiatry for the data-driven identification of brain-based subtypes. Biol Psychiatry. 2023;93:704–16. 10.1016/j.biopsych.2022.12.020 PMC10038896 36841702

[9] Litten RZ, Ryan ML, Falk DE, Reilly M, Fertig JB, Koob GF. Heterogeneity of alcohol use disorder: understanding mechanisms to advance personalized treatment. Alcoholism: Clin Exp Res. 2015;39:579–84. 10.1111/acer.12669 25833016

[10] Carroll KM. The profound heterogeneity of substance use disorders: implications for treatment development. Curr Dir Psychol Sci. 2021;30:358–64. 10.1177/09637214211026984 PMC8415637 34483503

[11] Donato S, Ray LA. Neurobiology and the treatment of alcohol use disorder: a review of the evidence base. Subst Abuse Rehabil. 2023;14:157–66. 10.2147/SAR.S409943 PMC10657770 38026786

[12] Gao CX, Dwyer D, Zhu Y, Smith CL, Du L, Filia KM, et al. An overview of clustering methods with guidelines for application in mental health research. Psychiatry Res. 2023;327:115265 10.1016/j.psychres.2023.115265 37348404

[13] Leggio L, Kenna GA, Fenton M, Bonenfant E, Swift RM. Typologies of alcohol dependence. from jellinek to genetics and beyond. Neuropsychol Rev. 2009;19:115–29. 10.1007/s11065-008-9080-z 19184441

[14] Drossel G, Brucar LR, Rawls E, Hendrickson TJ, Zilverstand A. Subtypes in addiction and their neurobehavioral profiles across three functional domains. Transl Psychiatry. 2023;13:1 10.1038/s41398-023-02426-1 PMC10113211 37072391

[15] Mann K, Roos CR, Hoffmann S, Nakovics H, Leménager T, Heinz A, et al. Precision medicine in alcohol dependence: a controlled trial testing pharmacotherapy response among reward and relief drinking phenotypes. Neuropsychopharmacol. 2018;43:891–9. 10.1038/npp.2017.282 PMC5809801 29154368

[16] Brucar LR, Rawls E, Haynos AF, Peterson CB, Zilverstand A. Mechanism-based subtyping in binge eating: understanding neurobehavioral heterogeneity across negative emotionality, approach behavior, and executive function. Transl Psychiatry. 2025;15:193 10.1038/s41398-025-03408-1 PMC12144091 40480995

[17] Koob GF, Volkow ND. Neurobiology of addiction: a neurocircuitry analysis. Lancet Psychiatry. 2016;3:760–73. 10.1016/S2215-0366(16)00104-8 PMC6135092 27475769

[18] Voon V, Grodin E, Mandali A, Morris L, Doñamayor N, Weidacker K, et al. Addictions neuroimaging assessment (ANIA): towards an integrative framework for alcohol use disorder. Neurosci Biobehav Rev. 2020;113:492–506. 10.1016/j.neubiorev.2020.04.004 32298710

[19] Zilverstand A, Huang AS, Alia-Klein N, Goldstein RZ. Neuroimaging impaired response inhibition and salience attribution in human drug addiction: a systematic review. Neuron. 2018;98:886–903. 10.1016/j.neuron.2018.03.048 PMC5995133 29879391

[20] Rodríguez GC, Russell MA, Claus ED. Systematic review on resting-state fMRI in people with AUD and people who binge drink. Mol Psychiatry. 2025;30:752–62. 10.1038/s41380-024-02796-y 39448806

[21] Bottino M, Bocková N, Poller NW, Smolka MN, Böhmer J, Walter H, et al. Relating functional connectivity and alcohol use disorder: a systematic review and derivation of relevance maps for regions and connections. Hum Brain Mapp. 2025;46:e70156 10.1002/hbm.70156 PMC11803412 39917866

[22] Steele VR, Ding X, Ross TJ Chapter 6 - Addiction: Informing drug abuse interventions with brain networks. In: Munsell BC, Wu G, Bonilha L, Laurienti PJ, editors. Connectomics. Academic Press; 2019 pp. 101–22. (The Elsevier and MICCAI Society Book Series). https://www.sciencedirect.com/science/article/pii/B9780128138380000066.

[23] Dupuy M, Chanraud S. Imaging the addicted brain: alcohol. Int Rev Neurobiol. 2016;129:1–31. 10.1016/bs.irn.2016.04.003 27503446

[24] Müller-Oehring EM, Jung YC, Pfefferbaum A, Sullivan EV, Schulte T. The resting brain of alcoholics. Cereb Cortex. 2015;25:4155–68. 10.1093/cercor/bhu134 PMC4816777 24935777

[25] Marquand AF, Wolfers T, Dinga R Phenomapping: methods and measures for deconstructing diagnosis in psychiatry. In: Passos IC, Mwangi B, Kapczinski F, editors. Personalized Psychiatry: Big Data Analytics in Mental Health. Cham: Springer International Publishing; 2019 pp. 119–34. 10.1007/978-3-030-03553-2_7.

[26] Wen J, Varol E, Yang Z, Hwang G, Dwyer D, Kazerooni AF, et al. Subtyping brain diseases from imaging data. In: Colliot O, editor. Machine Learning for Brain Disorders. New York, NY: Springer US; 2023 pp. 491–510. 10.1007/978-1-0716-3195-9_16. 37988509

[27] Nour MM, Liu Y, Dolan RJ. Functional neuroimaging in psychiatry and the case for failing better. Neuron. 2022;110:2524–44. 10.1016/j.neuron.2022.07.005 35981525

[28] Marquand AF, Wolfers T, Mennes M, Buitelaar J, Beckmann CF. Beyond lumping and splitting: a review of computational approaches for stratifying psychiatric disorders. Biol Psychiatry: Cognit Neurosci Neuroimaging. 2016;1:433–47. 10.1016/j.bpsc.2016.04.002 PMC5013873 27642641

[29] Drysdale AT, Grosenick L, Downar J, Dunlop K, Mansouri F, Meng Y, et al. Resting-state connectivity biomarkers define neurophysiological subtypes of depression. Nat Med. 2017;23:1 10.1038/nm.4246 PMC5624035 27918562

[30] Tozzi L, Zhang X, Pines A, Olmsted AM, Zhai ES, Anene ET, et al. Personalized brain circuit scores identify clinically distinct biotypes in depression and anxiety. Nat Med. 2024;30:2076–87. 10.1038/s41591-024-03057-9 PMC11271415 38886626

[31] Zhu T, Becquey C, Chen Y, Lejuez CW, Li CSR, Bi J. Identifying alcohol misuse biotypes from neural connectivity markers and concurrent genetic associations. Transl Psychiatry. 2022;12:253 10.1038/s41398-022-01983-1 PMC9203552 35710901

[32] Varol E, Sotiras A, Davatzikos C. HYDRA: revealing heterogeneity of imaging and genetic patterns through a multiple max-margin discriminative analysis framework. Neuroimage. 2017;145:346–64. 10.1016/j.neuroimage.2016.02.041 PMC5408358 26923371

[33] Sporns O. Graph theory methods: applications in brain networks. Dialogues Clin Neurosci. 2018;20:111–21. 10.31887/DCNS.2018.20.2/osporns PMC6136126 30250388

[34] Van Essen DC, Smith SM, Barch DM, Behrens TEJ, Yacoub E, Ugurbil K. The WU-Minn human connectome project: an overview. NeuroImage. 2013;80:62–79. 10.1016/j.neuroimage.2013.05.041 PMC3724347 23684880

[35] Van Essen DC, Ugurbil K, Auerbach E, Barch D, Behrens TEJ, Bucholz R, et al. The human connectome project: a data acquisition perspective. NeuroImage. 2012;62:2222–31. 10.1016/j.neuroimage.2012.02.018 PMC3606888 22366334

[36] Glasser MF, Sotiropoulos SN, Wilson JA, Coalson TS, Fischl B, Andersson JL, et al. The minimal preprocessing pipelines for the human connectome project. Neuroimage. 2013;80:105–24. 10.1016/j.neuroimage.2013.04.127 PMC3720813 23668970

[37] Glasser MF, Coalson TS, Robinson EC, Hacker CD, Harwell J, Yacoub E, et al. A multi-modal parcellation of human cerebral cortex. Nature. 2016;536:7615 10.1038/nature18933 PMC4990127 27437579

[38] Hubert L, Arabie P. Comparing partitions. J Classification. 1985;2:193–218. 10.1007/BF01908075

[39] Liu T, Yu H, Blair RH. Stability estimation for unsupervised clustering: a review. WIREs Computational Stat. 2022;14:e1575 10.1002/wics.1575 PMC9787023 36583207

[40] Nichols TE, Holmes AP. Nonparametric permutation tests for functional neuroimaging: a primer with examples. Hum Brain Mapp. 2002;15:1–25. 10.1002/hbm.1058 PMC6871862 11747097

[41] Agelink van Rentergem JA, Deserno MK, Geurts HM. Validation strategies for subtypes in psychiatry: a systematic review of research on autism spectrum disorder. Clin Psychol Rev. 2021;87:102033 10.1016/j.cpr.2021.102033 33962352

[42] Rawls E, Kummerfeld E, Zilverstand A. An integrated multimodal model of alcohol use disorder generated by data-driven causal discovery analysis. Commun Biol. 2021;4:1–12. 10.1038/s42003-021-01955-z PMC8012376 33790384

[43] Guimerà R, Nunes Amaral LA. Functional cartography of complex metabolic networks. Nature. 2005;433:895–900. 10.1038/nature03288 PMC2175124 15729348

[44] Power JD, Schlaggar BL, Lessov-Schlaggar CN, Petersen SE. Evidence for hubs in human functional brain networks. Neuron. 2013;79:798–813. 10.1016/j.neuron.2013.07.035 PMC3838673 23972601

[45] Bullmore E, Sporns O. Complex brain networks: graph theoretical analysis of structural and functional systems. Nat Rev Neurosci. 2009;10:3 10.1038/nrn2575 19190637

[46] Cao M, Wang Z, He Y. Connectomics in psychiatric research: advances and applications. Neuropsychiatr Dis Treat. 2015;11:2801–10. 10.2147/NDT.S63470 PMC4631424 26604764

[47] Wang X, Manza P, Li X, Ramos-Rolón A, Hager N, Wang GJ, et al. Reduced brain network segregation in alcohol use disorder: associations with neurocognition. Addiction Biol. 2024;29:e13446 10.1111/adb.13446 PMC11649955 39686721

[48] Luo X, Guo L, Dai XJ, Wang Q, Zhu W, Miao X, et al. Abnormal intrinsic functional hubs in alcohol dependence: evidence from a voxelwise degree centrality analysis. NDT. 2017;13:2011–20. 10.2147/NDT.S142742 PMC5546828 28814870

[49] Bordier C, Weil G, Bach P, Scuppa G, Nicolini C, Forcellini G, et al. Increased network centrality of the anterior insula in early abstinence from alcohol. Addiction Biol. 2022;27:e13096 10.1111/adb.13096 PMC9286046 34467604

[50] Herman AM, Critchley HD, Duka T. Trait impulsivity associated with altered resting-state functional connectivity within the somatomotor network. Front Behav Neurosci. 2020;14:111 10.3389/fnbeh.2020.00111 PMC7326939 32670033

[51] Herman AM, Duka T. Facets of impulsivity and alcohol use: what role do emotions play? Neurosci Biobehav Rev. 2019;106:202–16. 10.1016/j.neubiorev.2018.08.011 30343823

[52] Zheng H, Zhai T, Lin X, Dong G, Yang Y, Yuan TF. The resting-state brain activity signatures for addictive disorders. Med. 2024;5:201–223.e6. 10.1016/j.medj.2024.01.008 PMC10939772 38359839

[53] Kebets V, Holmes AJ, Orban C, Tang S, Li J, Sun N, et al. Somatosensory-motor dysconnectivity spans multiple transdiagnostic dimensions of psychopathology. Biol Psychiatry. 2019;86:779–91. 10.1016/j.biopsych.2019.06.013 31515054

[54] Dugré JR, Potvin S. Investigating the impact of lumping heterogenous conduct problems: aggression and rule-breaking rely on distinct spontaneous brain activity. Eur Child Adolesc Psychiatry. 2025;34:1207–19. 10.1007/s00787-024-02557-w PMC11909054 39143190

[55] Fede SJ, Grodin EN, Dean SF, Diazgranados N, Momenan R. Resting state connectivity best predicts alcohol use severity in moderate to heavy alcohol users. NeuroImage: Clin. 2019;22:101782 10.1016/j.nicl.2019.101782 PMC6438989 30921611

[56] Fascher M, Nowaczynski S, Muehlhan M. Substance use disorders are characterised by increased voxel-wise intrinsic measures in sensorimotor cortices: An ALE meta-analysis. Neurosci Biobehav Rev. 2024;162:105712 10.1016/j.neubiorev.2024.105712 38733896

[57] Cuthbert BN, Insel TR. Toward the future of psychiatric diagnosis: the seven pillars of RDoC. BMC Med. 2013;11:126 10.1186/1741-7015-11-126 PMC3653747 23672542

[58] Savage JE, Dick DM. Internalizing and externalizing subtypes of alcohol misuse and their relation to drinking motives. Addictive Behav. 2023;136:107461 10.1016/j.addbeh.2022.107461 36063573

[59] Hardee JE, Cope LM, Martz ME, Heitzeg MM. Review of neurobiological influences on externalizing and internalizing pathways to alcohol use disorder. Curr Behav Neurosci Rep. 2018;5:249–62. 10.1007/s40473-018-0166-5 PMC6876851 31768306

[60] Tolomeo S, Yu R. Brain network dysfunctions in addiction: a meta-analysis of resting-state functional connectivity. Transl Psychiatry. 2022;12:1–11. 10.1038/s41398-022-01792-6 PMC8799706 35091540

[61] Catani M, Dell’Acqua F, Thiebaut de Schotten M. A revised limbic system model for memory, emotion and behaviour. Neurosci Biobehav Rev. 2013;37:1724–37. 10.1016/j.neubiorev.2013.07.001 23850593

[62] Menon V. Large-scale brain networks and psychopathology: a unifying triple network model. Trends Cognit Sci. 2011;15:483–506. 10.1016/j.tics.2011.08.003 21908230

[63] Kwako LE, Bickel WK, Goldman D. Addiction biomarkers: dimensional approaches to understanding addiction. Trends Mol Med. 2018;24:121–8. 10.1016/j.molmed.2017.12.007 PMC6233300 29307501

[64] Bickel WK, Mellis AM, Snider SE, Athamneh LN, Stein JS, Pope DA. 21st century neurobehavioral theories of decision making in addiction: Review and evaluation. Pharmacol Biochem Behav. 2018;164:4–21. 10.1016/j.pbb.2017.09.009 PMC5747999 28942119

[65] Koob GF. The dark side of emotion: The addiction perspective. Eur J Pharmacol. 2015;753:73–87. 10.1016/j.ejphar.2014.11.044 PMC4380644 25583178

[66] DeMartini KS, Gueorguieva R, Pearlson G, Krishnan-Sarin S, Anticevic A, Ji LJ, et al. Mapping data-driven individualized neurobehavioral phenotypes in heavy alcohol drinkers. Alcoholism: Clin Exp Res. 2021;45:841–53. 10.1111/acer.14580 PMC13037707 33605439

[67] Witkiewitz K, Roos CR, Mann K, Kranzler HR. Advancing precision medicine for alcohol use disorder: replication and extension of reward drinking as a predictor of naltrexone response. Alcohol Clin Exp Res. 2019;43:2395–405. 10.1111/acer.14183 PMC6824945 31436886

[68] Brucar LR, Drossel G, Garza-Villarreal EA, Zilverstand A. Subtypes of cocaine use disorder and their neurobehavioral profiles. Transl Psychiatry. 2025;16:21 10.1038/s41398-025-03739-z PMC12804824 41266358

[69] Bzdok D, Meyer-Lindenberg A. Machine learning for precision psychiatry: opportunities and challenges. Biol Psychiatry: Cognit Neurosci Neuroimaging. 2018;3:223–30. 10.1016/j.bpsc.2017.11.007 29486863

[70] Williams LM, Carpenter WT, Carretta C, Papanastasiou E, Vaidyanathan U. Precision psychiatry and research domain criteria: implications for clinical trials and future practice. CNS Spectr. 2024;29:26–39. 10.1017/S1092852923002420 37675453

[71] Williams LM, Whitfield Gabrieli S. Neuroimaging for precision medicine in psychiatry. Neuropsychopharmacol. 2025;50:246–57. 10.1038/s41386-024-01917-z PMC11525658 39039140

[72] Drossel G, Zilverstand A. Towards personalized medicine: subtyping using functional profiles. Neuropsychopharmacology. 2024;49:347–8. 10.1038/s41386-023-01704-2 PMC10700316 37580461

[73] Manchia M, Pisanu C, Squassina A, Carpiniello B. Challenges and future prospects of precision medicine in psychiatry. Pharmgenomics Pers Med. 2020;13:127–40. 10.2147/PGPM.S198225 PMC7186890 32425581

[74] Ray LA, Grodin EN, Leggio L, Bechtholt AJ, Becker H, Feldstein Ewing SW, et al. The future of translational research on alcohol use disorder. Addiction Biol. 2021;26:e12903 10.1111/adb.12903 PMC7554164 32286721

[75] Baldwin H, Loebel-Davidsohn L, Oliver D, Salazar de Pablo G, Stahl D, Riper H, et al. Real-world implementation of precision psychiatry: a systematic review of barriers and facilitators. Brain Sci. 2022;12:934 10.3390/brainsci12070934 PMC9313345 35884740

[76] Song S, Zilverstand A, Gui W, Pan X, Zhou X. Reducing craving and consumption in individuals with drug addiction, obesity or overeating through neuromodulation intervention: a systematic review and meta-analysis of its follow-up effects. Addiction. 2022;117:1242–55. 10.1111/add.15686 34514666

[77] Song S, Zilverstand A, Gui W, Li HJ, Zhou X. Effects of single-session versus multi-session non-invasive brain stimulation on craving and consumption in individuals with drug addiction, eating disorders or obesity: a meta-analysis. Brain Stimul. 2019;12:606–18. 10.1016/j.brs.2018.12.975 30612944

[78] Soleimani G, Joutsa J, Moussawi K, Siddiqi SH, Kuplicki R, Bikson M, et al. Converging evidence for frontopolar cortex as a target for neuromodulation in addiction treatment. AJP. 2024;181:100–14. 10.1176/appi.ajp.20221022 PMC11318367 38018143

[79] Mehta DD, Praecht A, Ward HB, Sanches M, Sorkhou M, Tang VM, et al. A systematic review and meta-analysis of neuromodulation therapies for substance use disorders. Neuropsychopharmacology. 2024;49:649–80. 10.1038/s41386-023-01776-0 PMC10876556 38086901

[80] DiClemente CC, Corno CM, Graydon MM, Wiprovnick AE, Knoblach DJ. Motivational interviewing, enhancement, and brief interventions over the last decade: a review of reviews of efficacy and effectiveness. Psychol Addictive Behav. 2017;31:862–87. 10.1037/adb0000318 29199843

[81] Bickel WK, Athamneh LN, Snider SE, Craft WH, DeHart WB, Kaplan BA, et al. Reinforcer pathology: implications for substance abuse intervention. Curr Top Behav Neurosci. 2020;47:139–62. 10.1007/7854_2020_145 32462615

[82] Hobbs JDJ, Kushner MG, Lee SS, Reardon SM, Maurer EW. Meta-analysis of supplemental treatment for depressive and anxiety disorders in patients being treated for alcohol dependence. Am J Addict. 2011;20:319–29. 10.1111/j.1521-0391.2011.00140.x PMC3124006 21679263

[83] Kushner MG, Maurer EW, Thuras P, Donahue C, Frye B, Menary KR, et al. Hybrid cognitive behavioral therapy versus relaxation training for co-occurring anxiety and alcohol disorder: a randomized clinical trial. J Consulting Clin Psychol. 2013;81:429–42. 10.1037/a0031301 PMC3756682 23276124

[84] Elliott ML, Knodt AR, Ireland D, Morris ML, Poulton R, Ramrakha S, et al. What is the test-retest reliability of common task-functional mri measures? new empirical evidence and a meta-analysis. Psychol Sci. 2020;31:792–806. 10.1177/0956797620916786 PMC7370246 32489141

[85] Gordon EM, Laumann TO, Gilmore AW, Newbold DJ, Greene DJ, Berg JJ, et al. Precision functional mapping of individual human brains. Neuron. 2017;95:791–807.e7. 10.1016/j.neuron.2017.07.011 PMC5576360 28757305

[86] Laumann TO, Gordon EM, Adeyemo B, Snyder AZ, Joo SJ, Chen MY, et al. Functional system and areal organization of a highly sampled individual human brain. Neuron. 2015;87:657–70. 10.1016/j.neuron.2015.06.037 PMC4642864 26212711

[87] Zuo XN, Xu T, Milham MP. Harnessing reliability for neuroscience research. Nat Hum Behav. 2019;3:8 10.1038/s41562-019-0655-x 31253883

[88] O’Connor D, Potler NV, Kovacs M, Xu T, Ai L, Pellman J, et al. The healthy brain network serial scanning initiative: a resource for evaluating inter-individual differences and their reliabilities across scan conditions and sessions. Gigascience. 2017;6:1–14. 10.1093/gigascience/giw011 PMC5466711 28369458

[89] Carvalho AF, Heilig M, Perez A, Probst C, Rehm J. Alcohol use disorders. Lancet. 2019;394:781–92. 10.1016/S0140-6736(19)31775-1 31478502

[90] Santos MP, Loreto BBL, von Diemen L, Goi PD Clinical staging of alcohol use disorder: proposal of a new stratified approach. Trends in Psychiatry and Psychotherapy. 2024;(Ahead of Print):0–0. 10.47626/2237-6089-2024-0927 39561402

[91] McLellan AT, Koob GF, Volkow ND. Preaddiction—a missing concept for treating substance use disorders. JAMA Psychiatry. 2022;79:749–51. 10.1001/jamapsychiatry.2022.1652 35793096

[92] Morris J, Boness CL, Witkiewitz K. A continuum model of alcohol use and problems can advance public health goals without undermining treatment agendas. reply to commentaries. Drugs: Education, Prev Policy. 2024;31:287–8. 10.1080/09687637.2023.2244658 PMC11164551 38863692

[93] Miller AP, Kuo SIC, Johnson EC, Tillman R, Brislin SJ, Dick DM, et al. Diagnostic criteria for identifying individuals at high risk of progression from mild or moderate to severe alcohol use disorder. JAMA Netw Open. 2023;6:e2337192 10.1001/jamanetworkopen.2023.37192 PMC10565602 37815828

[94] Maxwell AM, Brucar LR, Zilverstand A. A systematic review of sex/gender differences in the multi-dimensional neurobiological mechanisms in addiction and their relevance to impulsivity. Curr Addict Rep. 2023;10:770–92. 10.1007/s40429-023-00529-9 PMC11395779 39282614

[95] Ge R, Sassi R, Yatham LN, Frangou S. Neuroimaging profiling identifies distinct brain maturational subtypes of youth with mood and anxiety disorders. Mol Psychiatry. 2023;28:1072–8. 10.1038/s41380-022-01925-9 PMC10005933 36577839

[96] Fu CHY, Antoniades M, Erus G, Garcia JA, Fan Y, Arnone D, et al. Neuroanatomical dimensions in medication-free individuals with major depressive disorder and treatment response to SSRI antidepressant medications or placebo. Nat Ment Health. 2024;2:164–76. 10.1038/s44220-023-00187-w PMC11211072 38948238

[97] Kaczkurkin AN, Sotiras A, Baller EB, Barzilay R, Calkins ME, Chand GB, et al. Neurostructural heterogeneity in youths with internalizing symptoms. Biol Psychiatry. 2020;87:473–82. 10.1016/j.biopsych.2019.09.005 PMC7007843 31690494

[98] Lalousis PA, Schmaal L, Wood SJ, Reniers RLEP, Barnes NM, Chisholm K, et al. Neurobiologically based stratification of recent-onset depression and psychosis: identification of two distinct transdiagnostic phenotypes. Biol Psychiatry. 2022;92:552–62. 10.1016/j.biopsych.2022.03.021 PMC10128104 35717212

