# Supplementary material for: Neurobiological subtypes in alcohol use disorder and their phenotypic and clinical profiles
Source: Mol Psychiatry. 2026 Jul 11;31(12):6868–80. doi: 10.1038/s41380-026-03748-4 (PMC13736515; doi:10.1038/s41380-026-03748-4)
Supplement: Supplementary file 1 [file 41380_2026_3748_MOESM1_ESM.pdf]

Neurobiological Subtypes in Alcohol Use Disorder and Their Phenotypic and Clinical Profiles

Leyla R. Brucar, PhD[1], Eric Rawls, PhD[1,2], and Anna Zilverstand, PhD[1,3*]
1. Department of Psychiatry & Behavioral Sciences, University of Minnesota, Minneapolis, MN, USA
2. Department of Psychology, University of North Carolina Wilmington, Wilmington, NC, USA
3. Medical Discovery Team on Addiction, University of Minnesota, Minneapolis, MN, USA

*Corresponding Author

Correspondence to Anna Zilverstand, PhD. 717 Delaware Street, Minneapolis, MN 55414
Email: annaz@umn.edu

Supplementary Materials

Supplementary Methods

1. Functional Connectivity Estimation and Graph Theory Metric Calculation
2. Evaluation of Participation Coefficient Between-Subject Variability and Test-Retest Reliability
3. Subtype Validation and Replicability
4. Analysis of Global Signal Variability
5. Internal Clinical Validation: Measures, Processing and Analysis
6. HYDRA-Derived Subtypes in a Combined AUD and AUD with Cannabis Dependence Sample

Supplementary Results

1. Evaluation of Participation Coefficient Between-Subject Variability and Test-Retest Reliability
2. Global Signal Variability
3. Internal Clinical Validation: Results of Exploratory Factor Analysis

Supplementary Figures

4. **Figure 1:** Overview of the Study Hypothesis
5. **Figure 2:** Schematic Summarizing the Study Methodological Pipeline
6. **Figure 3:** Schematic of Empirical Cluster and Graph Metric Selection
7. **Figure 4:** Results of Cluster and Graph Metric Selection
8. **Figure 5.** Variability and Test-Retest Reliability of Participation Coefficient in Controls and AUD
9. **Figure 6.** Differences in Global Signal Variability by Group
10. **Figure 7.** Parcel-Wise Beta-Map Similarity with and Without Global Signal Standard Deviation Adjustment
11. **Figure 8:** Results of Parallel Analysis used for Exploratory Factor Analysis
12. **Figure 9.** Validation of HYDRA-Derived Subtypes: Combined AUD/AUD-Cannabis Dependence Sample
13. **Figure 10:** Neuroimaging Signatures of HYDRA-Derived Subtypes: Combined AUD/AUD-Cannabis Dependence Sample
14. **Figure 11:** Subtype Phenotypic and Clinical Characterization: Combined AUD-Cannabis Dependence Sample

Supplementary References

The supplemental tables are contained in separate Excel files.

Supplementary Methods

1. Functional Connectivity Estimation and Graph Theory Metric Calculation

Functional connectivity matrices were constructed by extracting the mean BOLD timeseries for each parcel (379 parcels) and computing the z-transformed Pearson correlation coefficients between these time- courses and the time-courses of all other parcels. Both weighted and thresholded adjacency matrices were calculated. The thresholded matrices were proportionally thresholded at 15% - which is the median of an ideal cost range (approx. 0.01–0.30). This provides for optimized sparsity in the thresholded graph(1) and improved stability of measures as compared to absolute thresholds(2).

The brain connectivity toolbox (brain-connectivity-toolbox.net) was used to compute both weighted and thresholded graph metrics. We calculated five commonly used metrics that reveal meaningful information about crucial information processing patterns in the brain, specifically functional integration, segregation, centrality and modularity. Functional integration in the brain refers to the rapid combination of specialized information from distributed brain regions(3). Measures of integration estimate the ease with which brain regions communicate, often based on the concept of a path. Functional segregation in the brain pertains to the ability for specialized processing to occur within densely interconnected or neighboring groups of brain regions(3). Centrality measures a node's capacity to influence or be influenced by others based on its connection topology(3,4).

We calculated *Global Efficiency* as a measure of functional integration, quantifying the global information transfer between brain regions(5). Additionally, we computed *Local Efficiency* as a measure of functional segregation, quantifying local information transfer within smaller modules or specialized subnetworks in the brain(5). Two different measures of centrality were calculated, *Betweenness Centrality,* measuring the number of paths passing through a node, reflecting its importance in the network(3), and *Degree/Strength,* quantifying the number of links or neighbors a node has, reflecting its importance in the network(3). Participation coefficient(6) measures the number of links between a node and all other nodes in the modules of a graph. Once an optimal module assignment has been identified, each node's pattern of connections relative to this assignment can be quantified, its inter- and intra-module connections. Nodes with low participation coefficients, called ‘provincial hubs’, primarily link nodes within a single module, reflecting more segregative processing – specialized within-module processing(4,7). Nodes with higher participation coefficient, called ‘connector hubs’, predominantly link nodes across different modules, reflecting more integrative processing – global communication across the whole network(4,7). Module assignments for computing participation coefficient were chosen a priori based on the multimodal parcellation used to parcellate the brain(8) which additionally assigns each of the 360 cortical parcels into 22 networks/modules. We additionally grouped all 19 anatomically defined CIFTI subcortical areas into one module. In total, 23 modules (22 cortical, 1 subcortical) were used for calculating participation coefficient.

2. Evaluation of Participation Coefficient Between-Subject Variability and Test-Retest Reliability

To characterize the behavior of participation coefficient in Controls and AUD, we evaluated both between-subject variability and test–retest reliability in our groups. Parcel-wise variability (standard deviation across subjects within each parcel) was quantified as the standard deviation of participation coefficient across subjects within each group (REST1). Group differences in variability were assessed by (i) comparing the distribution of parcel-wise standard deviations between groups using the Wilcoxon rank-sum test and (ii) conducting parcel-wise Levene’s tests for equality of variance, with FDR correction applied across all parcellated brain regions.

Test–retest reliability was assessed in subjects with both REST1 and REST2 scans. For each parcel and group separately, the intraclass correlation coefficient (ICC) was computed using participation coefficient values from REST1 and REST2. Group differences in parcel-wise ICC distributions were evaluated using the Wilcoxon signed-rank test

3. Subtype Validation and Replicability

Resampling-Based Validation

The stability of the derived subtype solution in regard to sampling variability was evaluated with the following steps:

1. Randomly select 80% of the subjects from both the control and patient group, maintaining the original case-control ratio.
2. Run HYDRA on the subsampled data, including age and sex/gender as covariates and evaluating cluster solutions from 2 to 10 using 10-fold cross-validation, to obtain ARI values for each cluster solution assessed.
3. Repeat steps 1 and 2 for a total of 100 times
4. Evaluate the consistency of the optimal solution identified from the subsamples with the cluster solution obtained from the original, unperturbed data.

Permutation Testing

Statistical significance of the clustering solution was assessed with the following steps:

1. Randomly shuffle features within the control and patient groups.
2. Run HYDRA on the shuffled data (*i)*, including age and sex/gender as covariates and evaluating cluster solutions from 2 to 10 using 10-fold cross-validation, to obtain an ARI_*alternative_i*.
3. Repeat steps 1 and 2 a total of 285 times to generate a distribution of ARI_*alternative* values.
4. Compare the observed ARI from the main analysis (non-shuffled data) against this ARI_alternative distribution using a two-sample t-test.

If the obtained p-value is below the significance threshold (0.05), we concluded that the derived subtypes were statistically significant.

Temporal Stability

To assess the temporal stability of the derived HYDRA subtypes, we compared the clustering results from the REST1 acquisition (N = 668; Male = 281; AUD = 147; HC = 505), acquired on scan day 1 of the HCP protocol and used in the main analysis, to those from the REST2 acquisition (N = 646; Male = 275; AUD = 141; HC = 505), obtained on scan day 2 of the HCP protocol. The analysis involved the following steps:

HYDRA clustering on REST2 Data
- Following the same procedure as the main analysis, HYDRA was applied to the REST2 data acquisition, including age and sex/gender as covariates, evaluating cluster solutions from 2 to 10 using 10-fold cross-validation based on the ARI.

Assessment of Cluster Solution Similarity Between Acquisition Days
- *Model Similarity:* Overall similarity between the REST 1 and REST 2 cluster solutions was quantified with the ARI.

- *Model Dissimilarity:* Overall dissimilarity was calculated using the Mirkin Metric [B.G. Mirkin, 1996], quantifying cluster assignment mismatch probability.
- *Subtype Assignment Similarity:* The percentage overlap of individuals between the HYDRA subtypes derived from the REST1 and REST2 acquisitions.
- *Neurobiological Signature Similarity:* Evaluated whether the discriminant neurobiological signatures characterizing the subtypes were preserved between REST1 and REST2.

4. Analysis of Global Signal Variability

To evaluate whether individual differences in global signal (GS) intensity influenced our results, we calculated global signal standard deviation (GS SD) per subject and tested whether it differed between our AUD and Control groups used as input to HYDRA. We additionally conducted a supplementary analysis including GS SD as a covariate in our graph theory analysis between the derived subtypes and controls. All other analysis specifications, covariates, and multiple-comparison correction procedures were identical to those used in the primary analyses (without GS SD adjustment). We then compared results from analyses with and without GS SD to evaluate its impact.

5. Internal Clinical Validation: Measures, Processing and Analysis

A comprehensive set of behavioral and self-report assessments and tasks were collected, spanning psychiatric and substance use history, personality, cognition, emotion, social function, and demographics For details see the Human Connectome Project(9) S1200 Release Reference Manual: https://www.humanconnectome.org/storage/app/media/documentation/s1200/HCP_S1200_Release_Reference_Manual.pdf

All available and complete phenotypic and behavioral task measures were used in the analysis for internal clinical validation of the derived subtypes (see **Supplementary Table 1** for complete list). When provided, we prioritized using summary scores encompassing the task-constructs of interest rather than single item-level scores. Additionally, if both age- and sex/gender-adjusted scores were available, we included only the adjusted scores.

Clinical use measures were evaluated to assess the clinical relevance of the derived subtypes, including alcohol and substance use characteristics. For a complete list of all clinical measures included, see **Supplementary Table 2**.

Phenotypic Data Processing: Exploratory Factor Analysis

We conducted an Exploratory Factor Analysis (EFA) on all available phenotypic/clinical and task measures (100 measures) to identify underlying latent factors while maintaining a comprehensive representation of the entire phenotypic space (as described in Rawls et al.(10)). Monte Carlo permutation analysis(11) (parallel analysis) was implemented to determine the number of factors to retain at a statistical significance of $p<.05$ (57). Latent factors were then extracted using maximum likelihood estimation with the expectation-maximization (EM) algorithm(12). We used 'direct oblimin' rotation to accommodate correlated factors, ensuring effective data reduction in a large variable space where factors are expected to be closely related but separable(12). To best operationally define the derived factors, factor loadings >0.3 were retained(12). Both the parallel analysis and EFA were conducted using the *'psych'* package in R. The derived EFA latent factors were subsequently used to evaluate the phenotypic profiles of the HYDRA subtypes.

Group Comparisons via Linear-Mixed Models

Comparisons on these indicators (i.e., derived EFA latent factors, use measures) were examined between groups using a series of linear mixed models (*lme4* package, R [v4.4.0, R Project for Statistical Computing]. Models included a fixed effect of group (cluster, controls), variable (measures included), group-by-variable interaction, and covariates (age, sex/gender). To account for sibling status, Family-ID was included as a random intercept, uniquely identifying each biological family as a categorical factor. Post-hoc analyses examining significant fixed effects were performed (*emmeans* package R [v4.4.0, R Project for Statistical Computing]), applying a 5% FDR correction across all comparisons

6. Replication of HYDRA-Derived Subtypes in a Combined AUD and AUD with Cannabis Dependence Sample

We evaluated whether the subtype solution when including a sample of individuals with comorbid AUD and Cannabis Dependence (AUD-CD) into the previous AUD group would generalize to, or meaningfully differ from, our identified AUD-ONLY subtype solution. To test this, we reran the entire HYDRA pipeline, including subtype identification and reproducibility analyses, using the original sample (N=668) and adding individuals with comorbid AUD and DSM-IV Cannabis Dependence (AUD-CD; N=55) to the AUD group. This yielded a total sample of N=723 (AUD/AUD-CD: 202; Controls: 521).

As with the original analysis, HYDRA semi-supervised clustering was applied to the AUD/AUD-CD, using participation coefficient as input, including age and sex/gender as covariates. The optimal number of clusters (k) was selected by evaluating cluster solutions from k = 2 to 10 using 10-fold cross-validation based on the Adjusted Rand Index. The cluster solution with the highest ARI, indicating the most stable solution, was selected for subsequent analyses.

Consistent with the original analysis pipeline, resampling-based validation was implemented (100 iterations using 80% stratified subsamples with replacement) to evaluate stability in relation to sampling variability, and permutation testing was conducted to assess statistical significance ($p<0.05$, two-tailed test). We further examined the overlap in cluster assignments between the original AUD-ONLY solution and the combined AUD/AUD-CD solution to better understand the consistency of subtype membership and the extent to which comorbid CD influenced cluster structure. Similarity in cluster assignment was evaluated using the ARI, calculated between solutions.

We conducted graph theory analysis to investigate whether the neuroimaging signatures observed in the AUD/AUD-CD sample were similar to those in the AUD-ONLY sample. Finally, we characterized phenotypic profiles by repeating the group comparisons analysis using linear mixed models with the derived phenotypic factors from the EFA.

Supplementary Results

1. Results of Participation Coefficient Between-Subject Variability and Test-Retest Reliability

To characterize the behavior of participation coefficient in the Control and AUD groups, we examined both between-subject variability and test–retest reliability. In REST1, parcel-wise variability did not differ between Controls and AUD (Wilcoxon rank-sum test comparing parcel-wise SD distributions: $p = 0.19$; Cliff's $\delta = -0.055$, 95% CI, 0.14-0.03). Moreover, Levene's tests identified no parcels surviving FDR correction for unequal variance between groups, indicating no systematic heteroskedasticity in Controls relative to the AUD group.

We next assessed test–retest reliability using ICC among subjects with both REST1 and REST2 scans. Participation coefficient demonstrated modest reliability in both groups. Mean parcel-wise ICC was 0.38 in Controls and 0.39 in AUD. The distribution of parcel-wise ICC values did not differ significantly between groups (Wilcoxon signed-rank test: $p = 0.29$; Cliff's $\delta = 0.044$; 95% CI: −0.040-0.13). Together, these findings indicate that participation coefficient exhibits comparable levels of between-subject variability and test–retest stability in amongst our groups. Thus, the derived HYDRA subtype solution is unlikely to be driven by systematic differences in measurement variance or reliability between the AUD and Control groups.

2. Results of Global Signal Variability Analysis

Whole-brain variation in global signal intensity (GS DS) did not differ between AUD and control groups ($\beta = -0.0046$, $t = -0.575$, $p = 0.57$, $R^2 < 0.001$; **Supplementary Figure 6**), indicating that between-group differences in global signal intensity are unlikely to account for the observed effects. We further evaluated the effect of global signal variance on HYDRA subtype comparisons by repeating the graph theory analysis including GS SD as a covariate. Although fewer regions survived whole-brain FDR correction after including GS SD, the patterns of subtype-related participation coefficient differences remained highly consistent with the primary analysis without GS SD adjustment (beta-map correlations: Subtype1 $r = 0.87$; Subtype2 $r = 0.96$; **Supplementary Figure 7**), supporting stable HYDRA-derived brain signatures. Specifically, HYDRA Subtype 1 was similarly characterized by increased participation coefficient in sensory-motor regions and decreases in frontoparietal, salience and subcortical regions, whereas HYDRA Subtype 2 exhibited the inverse pattern (**Supplementary File 1: Table 6**).

3. Internal Clinical Validation: Results of Exploratory Factor Analysis

Bartlett's test of Sphericity ($\chi^2 = 48774.45$, $p<.01$) and Kaiser–Meyer–Olkin test (KMO=0.87) confirmed data suitability for Exploratory Factor Analysis (EFA). The EFA identified 18 latent factors, reducing the initial 100-variable phenotypic space (**Supplementary Table 1**) to an 18-dimensional latent space (**Supplementary Figure 3**). Model fit indices indicated good factor separation and good fit (Root Mean Square Error of Approximation [RMSEA] = 0.03, Tucker-Lewis Index = 0.86) supporting the adequacy of the factor solution. Details on factor labels and loadings are provided in **Supplementary Table 10**. The latent factors were subsequently used for internal clinical validation, enabling phenotypic characterization of the neuroimaging-derived subtypes using deeply phenotyped, multimodal data that were independent of the data used in the clustering process (**Figure 3**; **Supplementary Table 11**).

Supplementary Figures

Supplementary Figure 1: Overview of the Study Hypothesis.
We hypothesized that multiple, distinct subtypes exist that underlie the neurobiological and clinical heterogeneity observed in Alcohol Use Disorder (AUD). We used HYDRA, a semi-supervised algorithm that clusters individuals within a ‘target’ group (i.e., AUD) based on their patterns of deviation from a fixed ‘reference’ group (i.e., controls), thereby minimize confounding variation. Using resting-state graph theory metrics as input, HYDRA was used to identify potential neurobiological subtypes of AUD. Subsequent analyses examined the clinical and phenotypic characteristics of these subtypes.

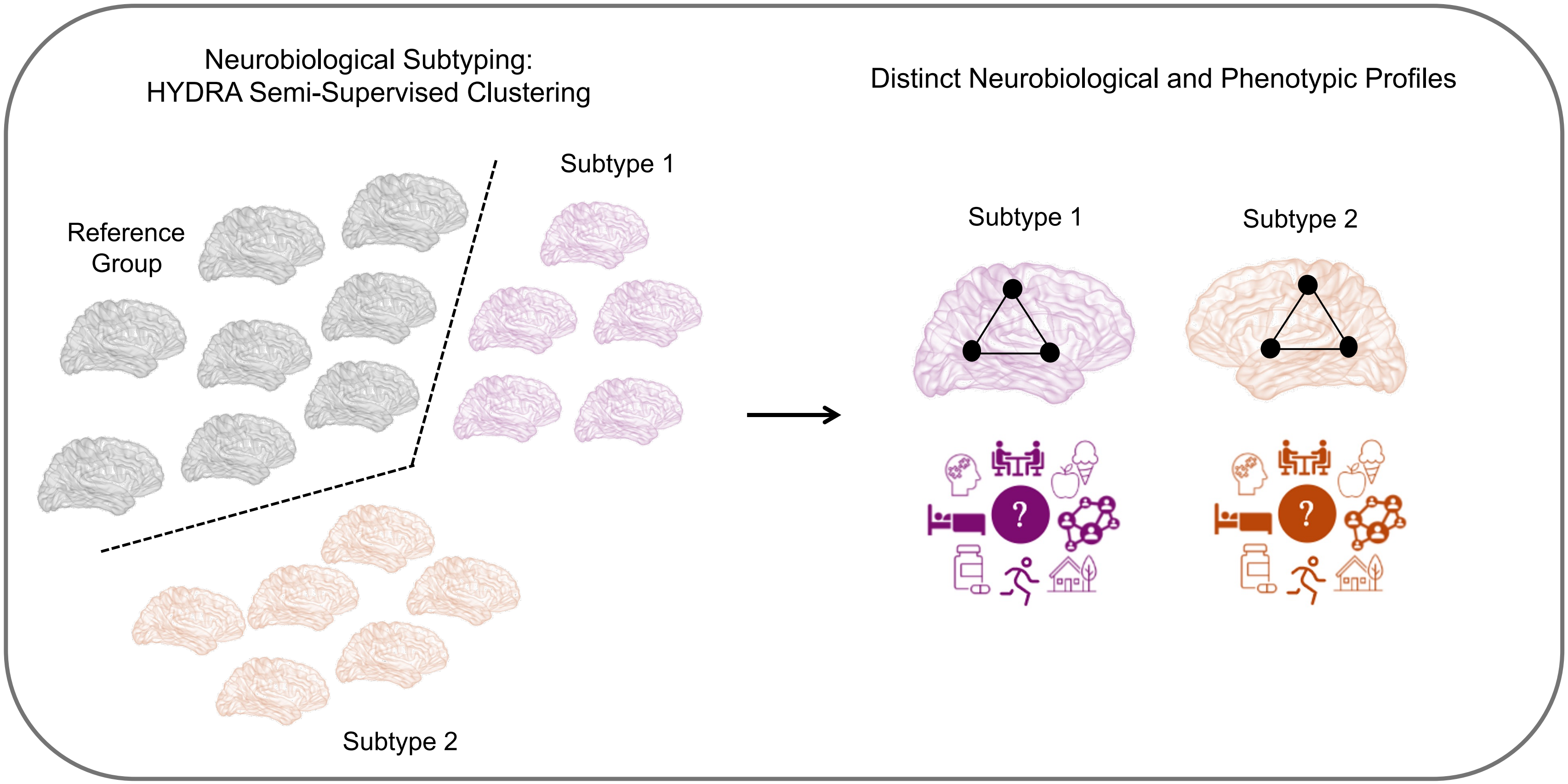

Supplementary Figure 2: Schematic Summarizing the Study Methodological Pipeline.
A. MRI processing, functional connectivity and graph metric calculation. **B.** Empirical cluster and graph metric selection via HYDRA semi-supervised clustering. **C.** Validation and replication of identified neurobiological subtypes. **D.** Subtype neurobiological and phenotypic characterization.

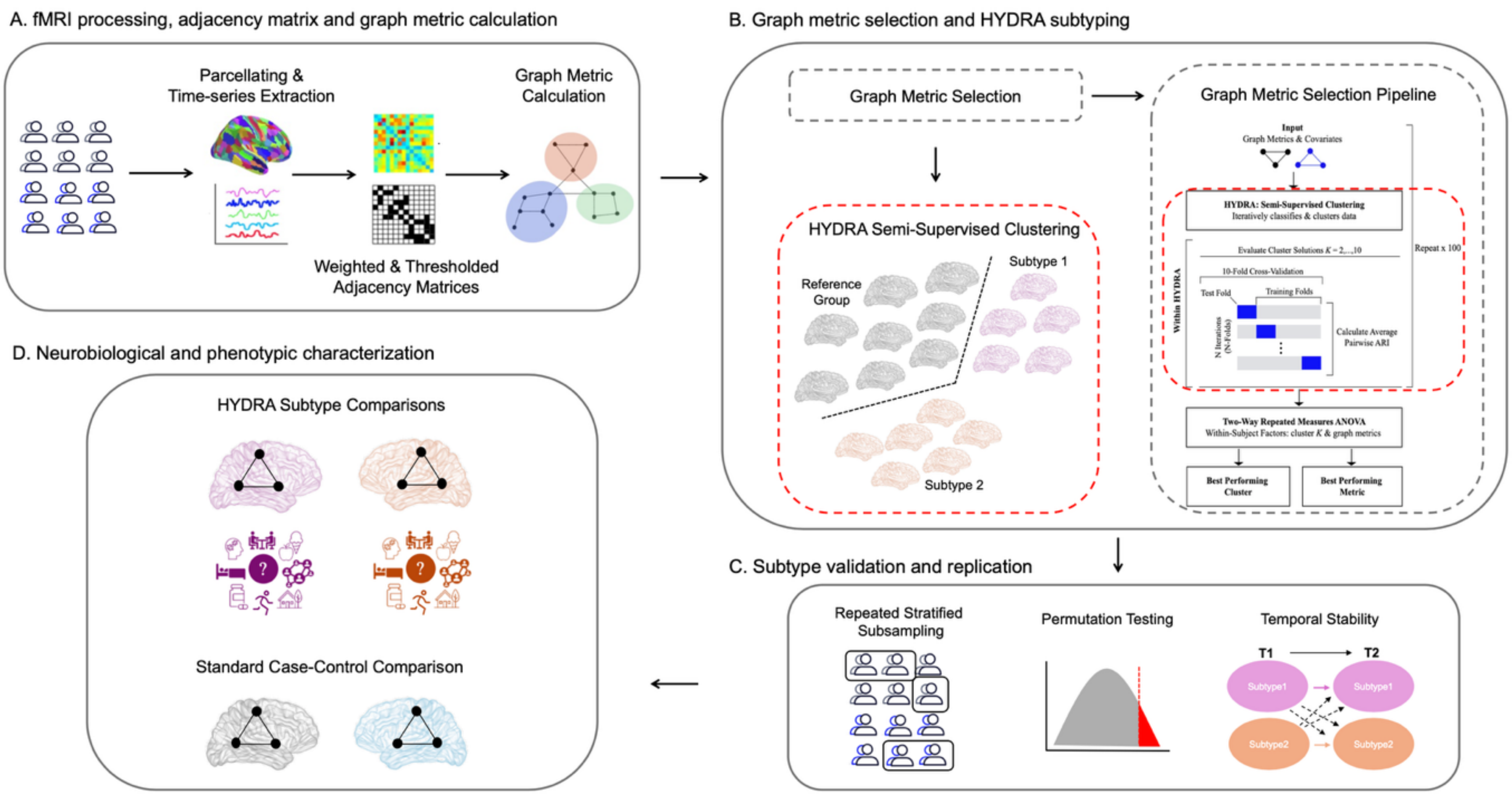

Supplementary Figure 3: Schematic of Empirical Cluster and Graph Metric Selection. Pipeline for cluster and graph metric selection. Each graph metric was used as input to HYDRA, assessing cluster solutions from $K = 2,\ldots,10$, using 10-fold cross validation based on the Adjusted Rand Index (ARI). Within each tested K, the ARI was computed between all pairwise folds. This was repeated 100 times for each graph metric, resulting in 100 ARI observations for each tested cluster solution and each metric. A two-way repeated measures ANOVA was used to empirically determine which cluster solution and metric was most reliable.

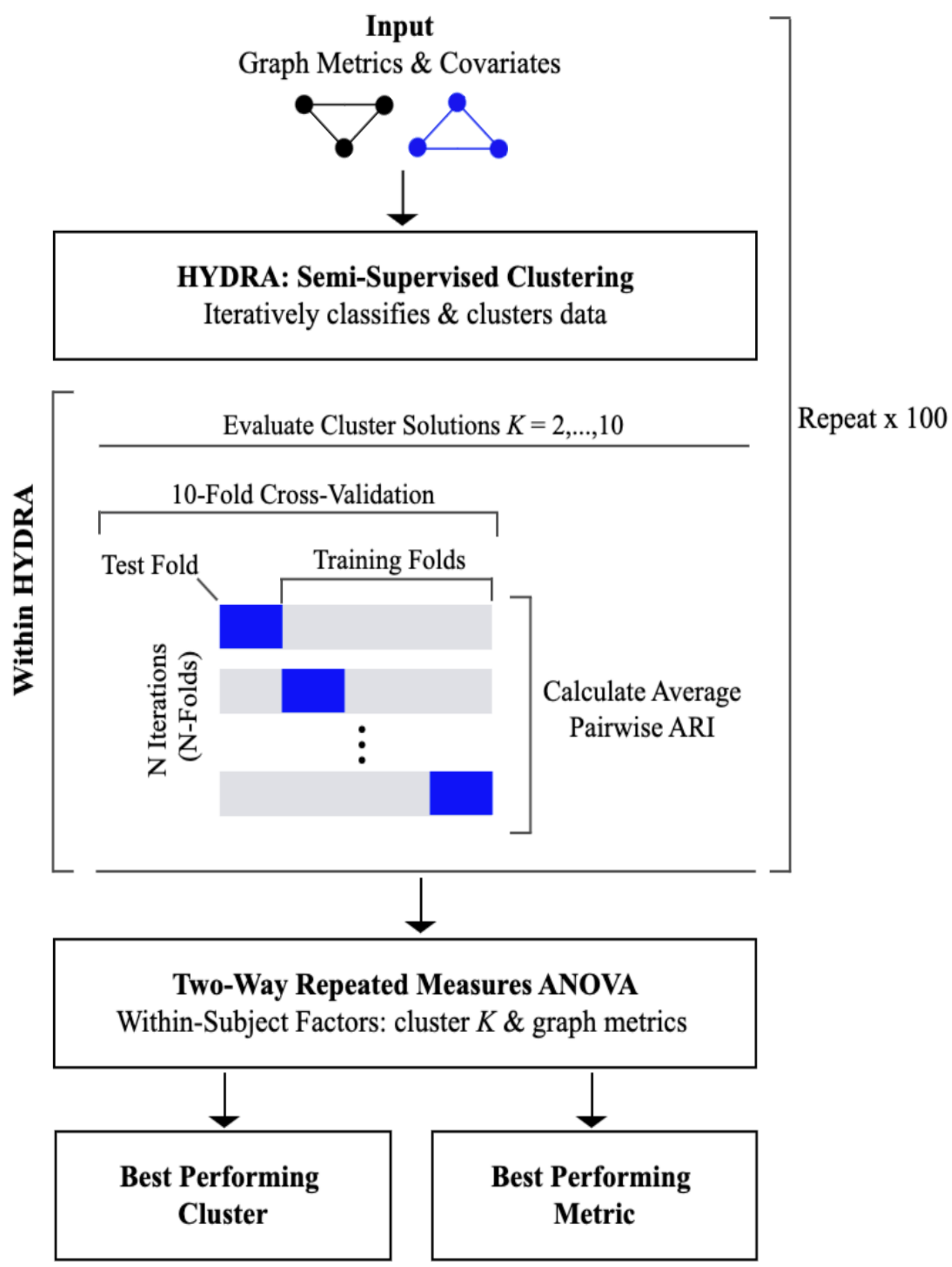

Supplementary Figure 4: Results of Cluster and Graph Metric Selection.
Cluster solutions (k = 2…10) were evaluated using 10-fold cross-validation and Adjusted Rand Index (ARI) across 100 iterations of HYDRA, per *k* and graph metric. A two-way repeated measures ANOVA assessed performance across cluster solutions and metrics. The cluster solution and graph metric with the highest average ARI, was chosen for subsequent analyses. **** $p\text{-}FDR < 0.0001$.

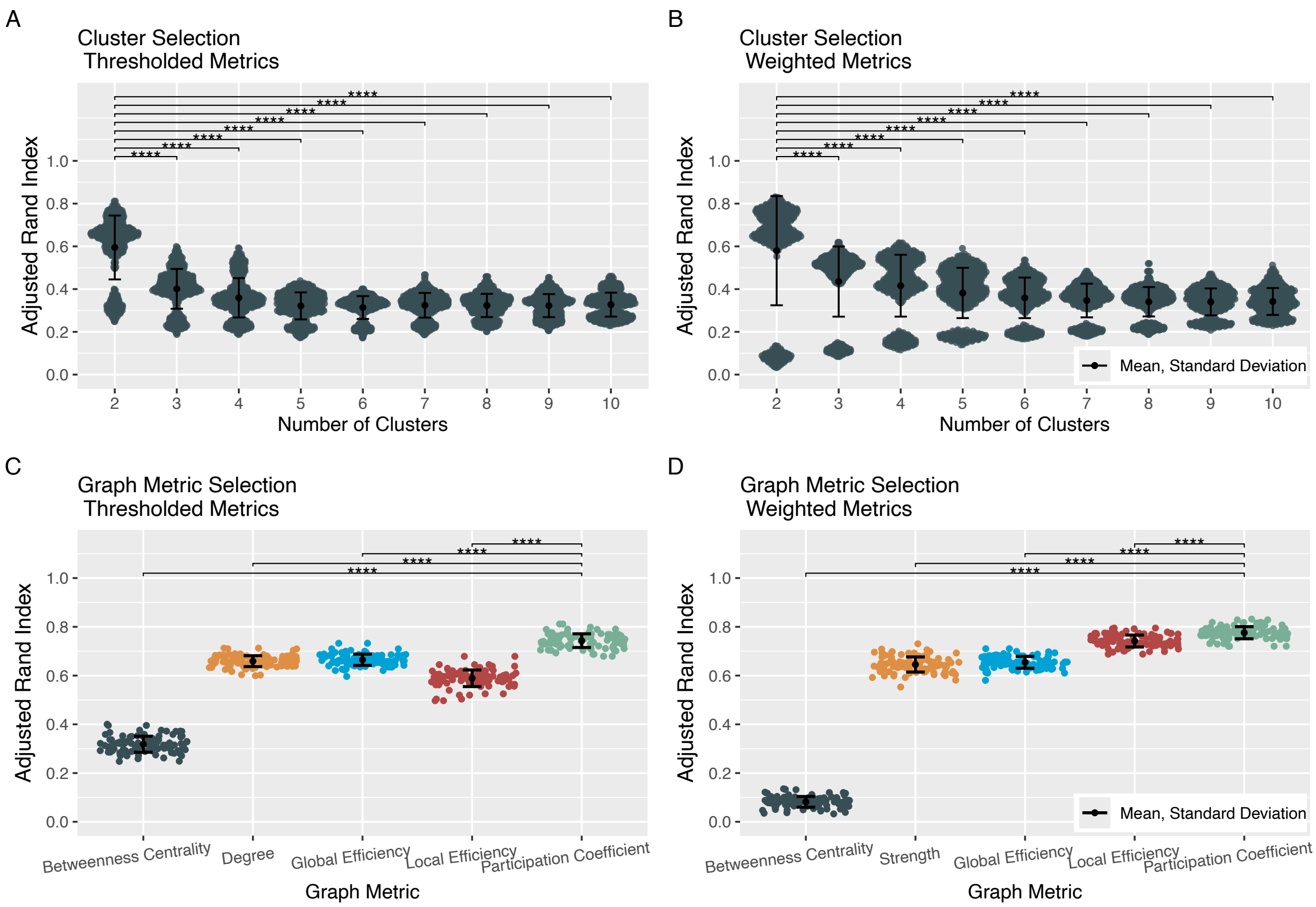

Supplementary Figure 5. Variability and Test-Retest Reliability of Participation Coefficient in Controls and AUD.

A. Parcel-wise between-subject variability in Scan Day 1 data (REST1) was evaluated between groups using the Wilcoxon rank-sum test, and equality of variance was assessed using parcel-wise Levene's test. Parcel-wise Levene's tests were FDR corrected for multiple comparisons. **B.** Parcel-wise test-retest reliability across Scan Day 1 and Scan Day 2 (REST1 vs REST2) was evaluated using intraclass correlation coefficients (ICCs) within each group, and group differences in the parcellated brain region ICC values were compared using the Wilcoxon signed-rank test.

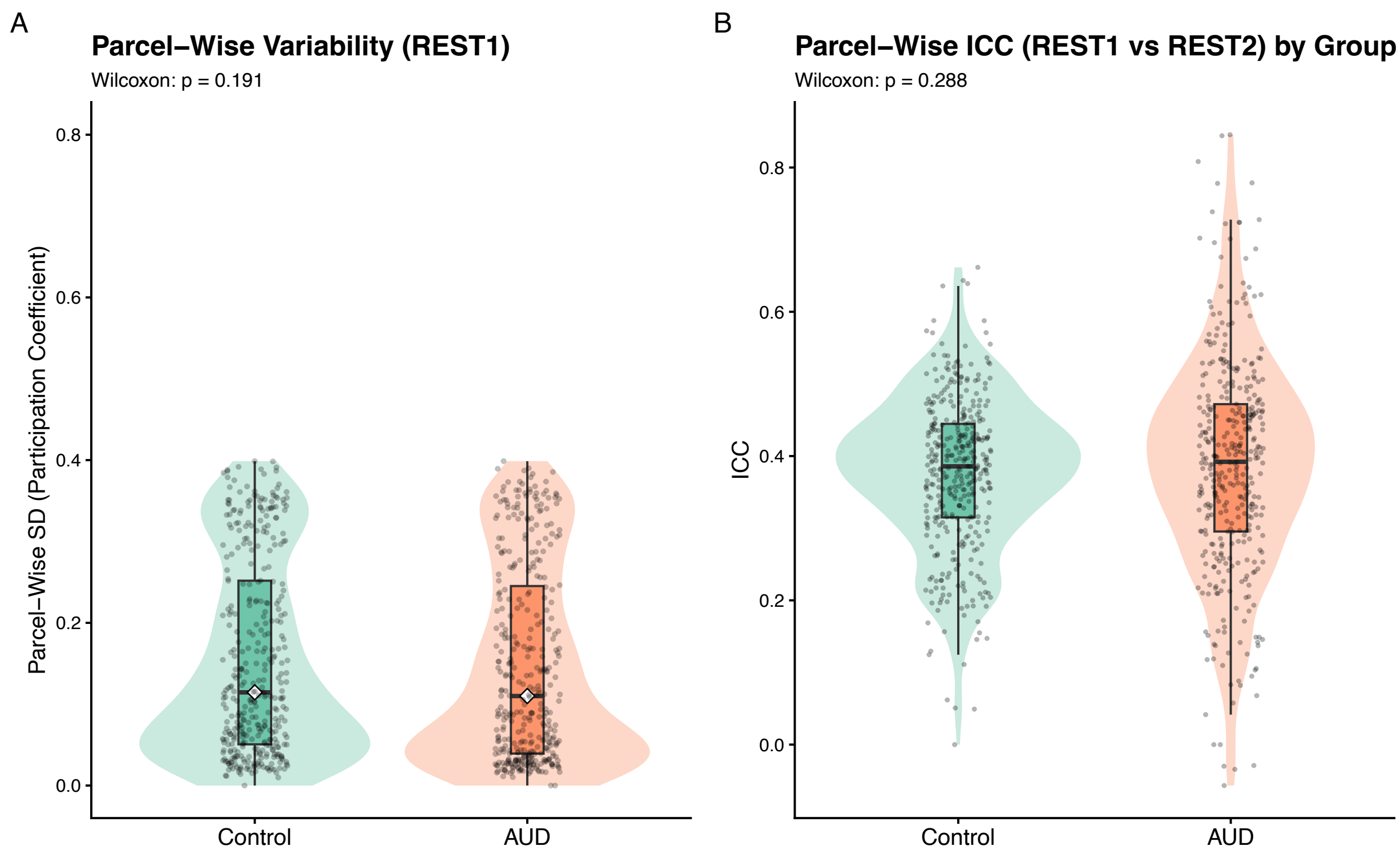

Supplementary Figure 6. Differences in Global Signal Variability by Group.
Histogram depicting the global signal standard deviation (GS DS) of the AUD and Control groups, which were used as input to HYDRA. Group differences in GS SD were not significant ($p = 0.566$).

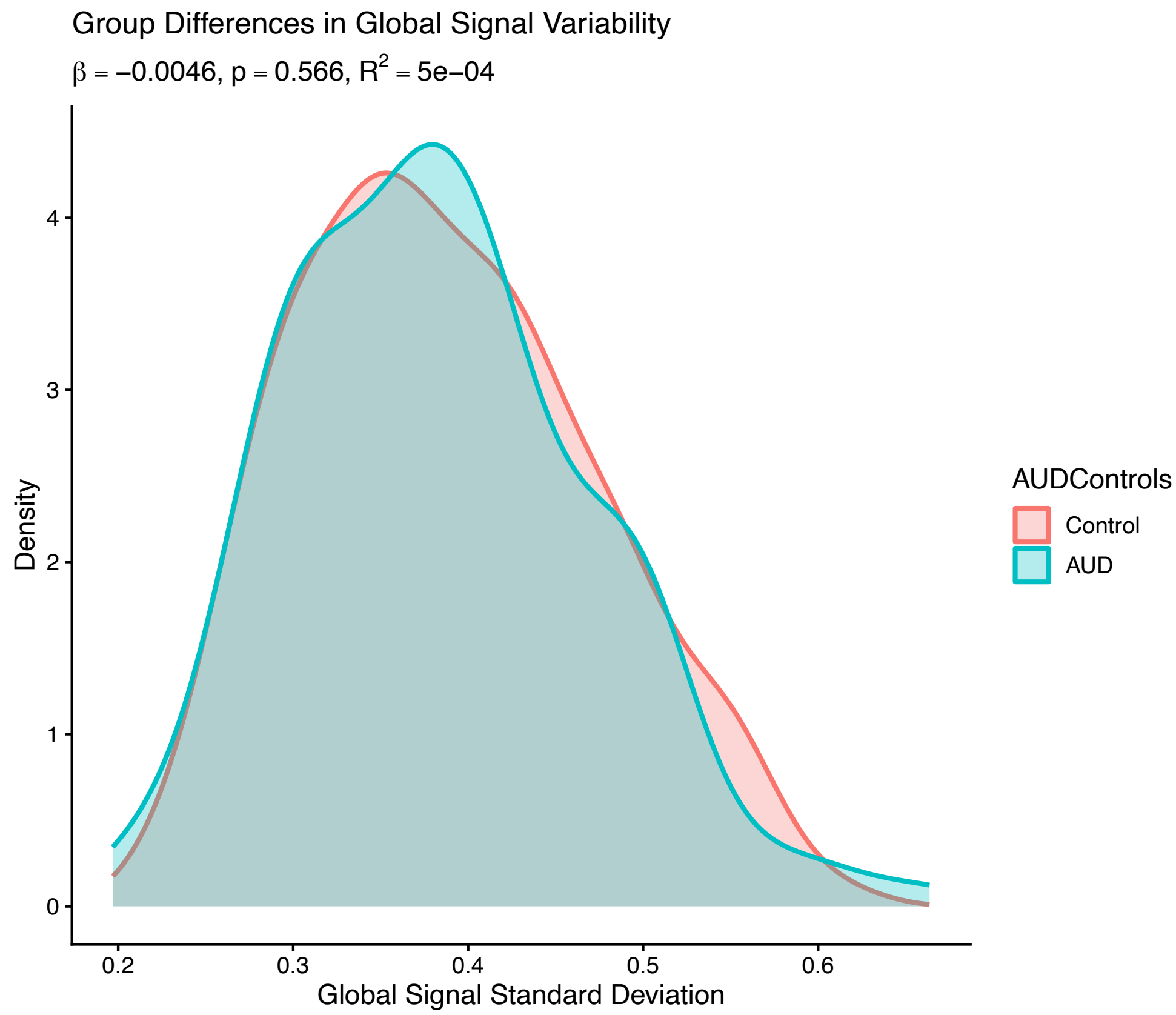

Supplementary Figure 7. Parcel-Wise Beta-Map Similarity with and without Global Signal Standard Deviation Covariate Adjustment.
Spatial patterns of parcel-wise participation coefficient difference in subtypes were highly consistent between analyses with and without global signal standard deviation adjustment. *GS SD,* Global signal standard deviation.

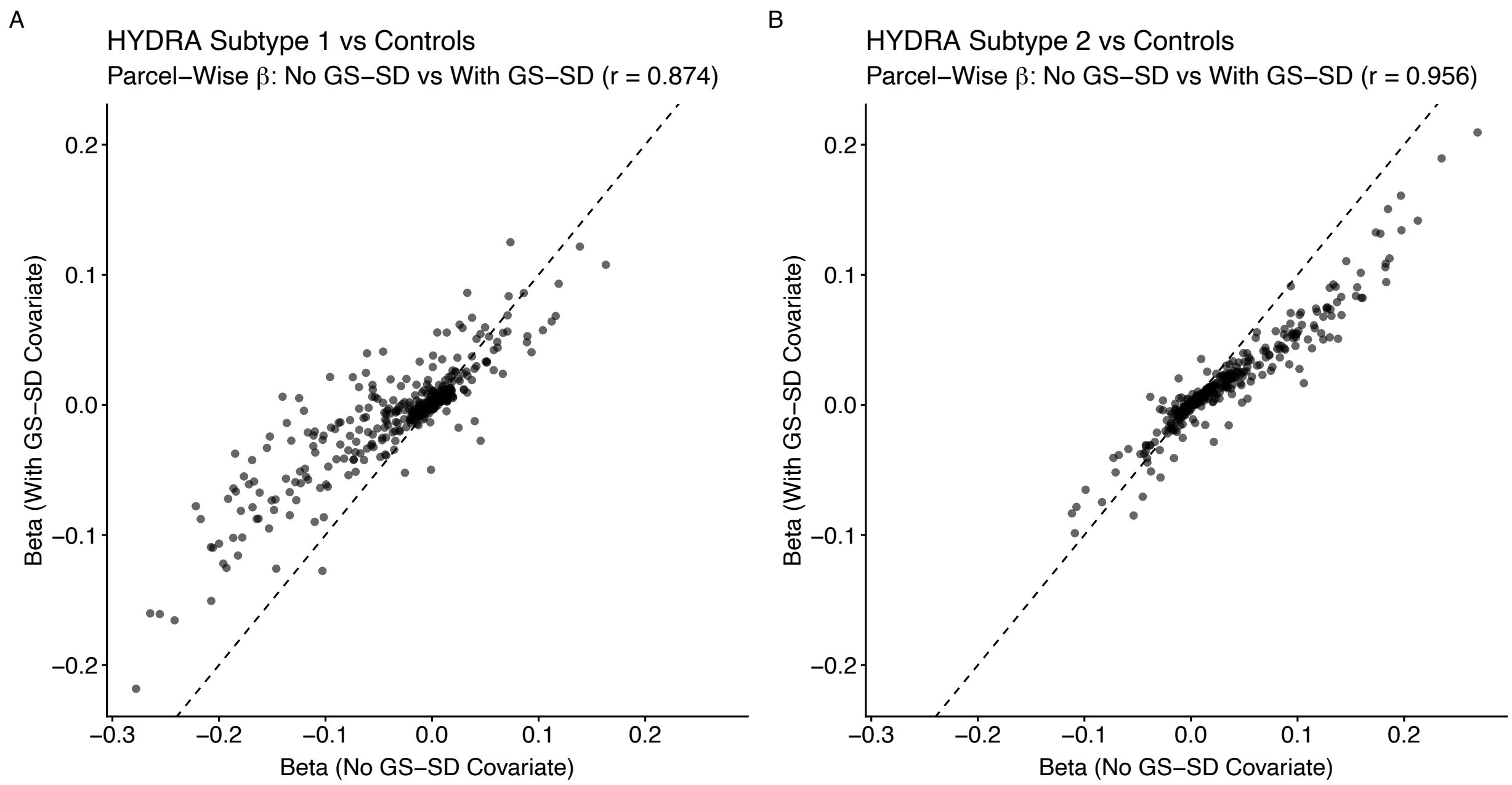

Supplementary Figure 8: Results of Parallel Analysis used for Exploratory Factor Analysis
Scree plot from permutation test of eigenvalue significance (parallel analysis) used to determine the number of factors in exploratory factor analysis. Latent factors were retained when their observed eigenvalues exceeded those of the corresponding factors derived from simulated (permuted) data.

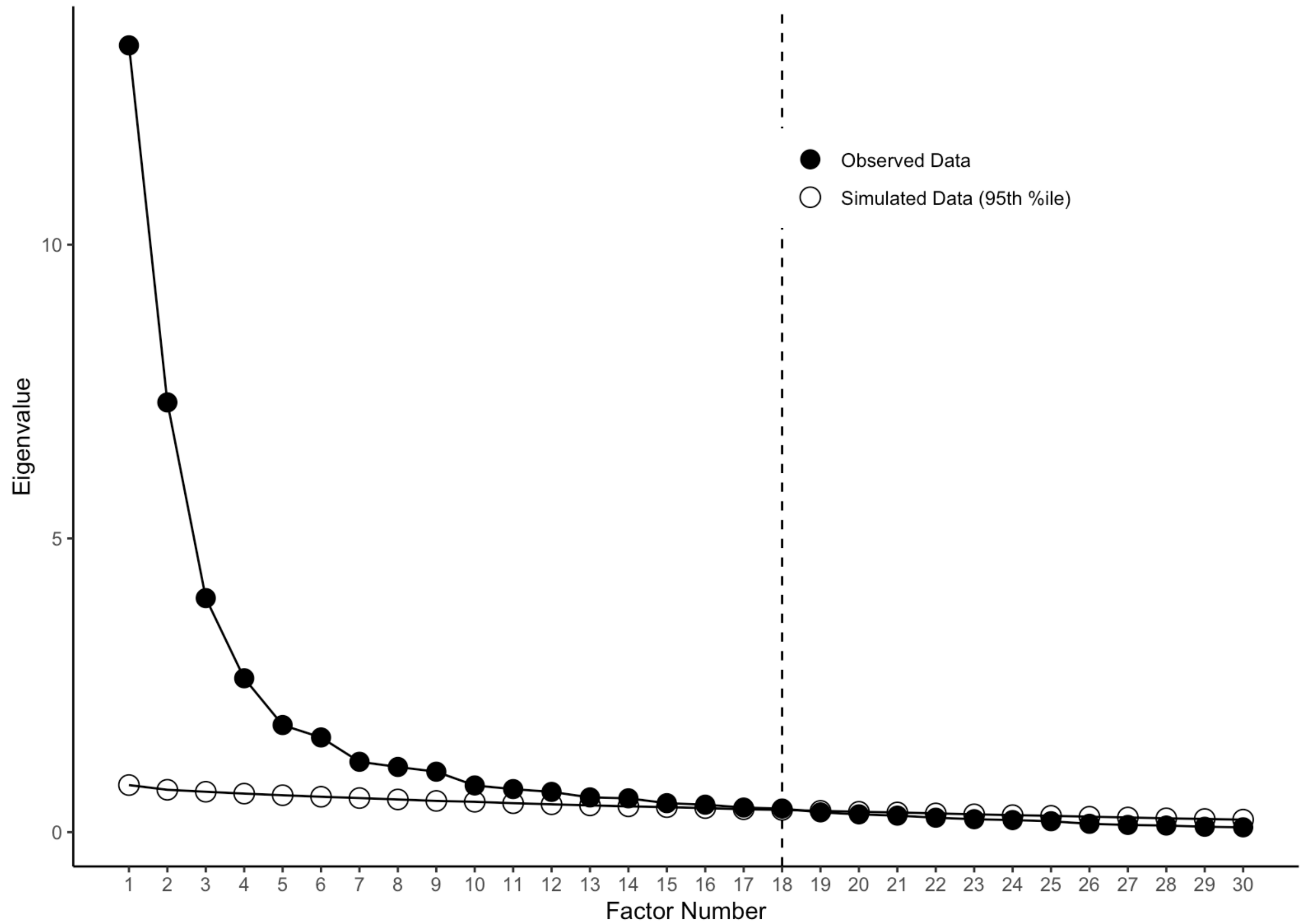

Supplementary Figure 9. Validation of HYDRA-Derived Subtypes: Combined AUD/AUD-Cannabis Dependence Sample.
A. Resampling Based Validation: Adjusted Rand Index (ARI) for clustering solutions ($k = 2,\ldots,10$), computed across 100 HYDRA iterations using 80% stratified subsampled with replacement. **B.** Permutation Test: ARI stability across 350 permuted samples (Cohen's d = 1.45; 95% CI, 1.30-1.60; p<.05). **C.** Assignment Stability: Stability of cluster assignments between the original AUD-ONLY subtype solution and the expanded combined AUD/AUD-CD subtype solution.

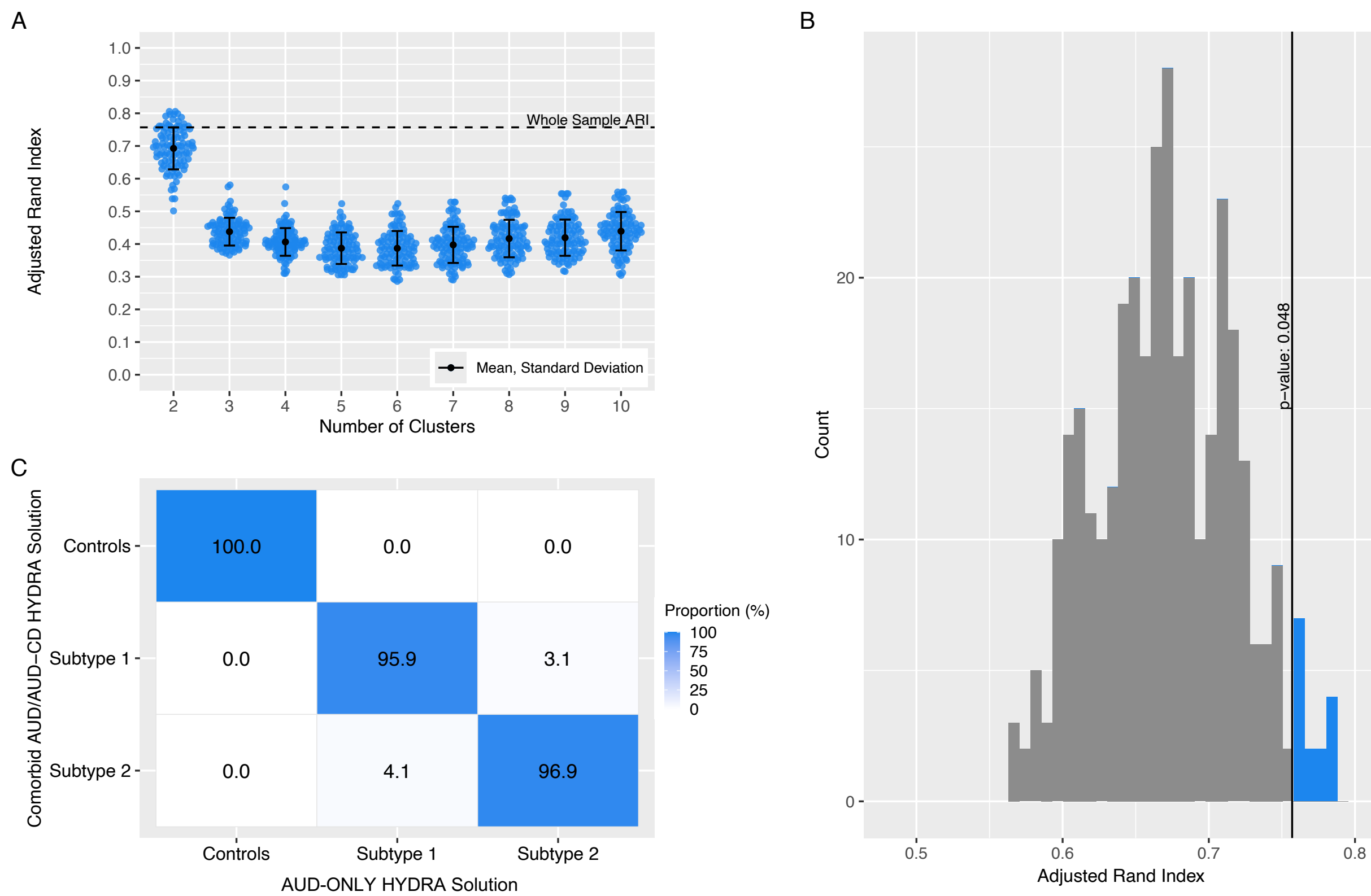

Supplementary Figure 10: Neuroimaging Signatures of HYDRA-Derived Subtypes: Combined AUD/AUD-Cannabis Dependence Sample.
Subtype-specific alterations in participation coefficient relative to controls. **A-B.** HYDRA-derived neuroimaging signatures from original AUD-ONLY sample: A. Subtype 1. B. Subtype 2. **C-D.** HYDRA-derived neuroimaging signatures from expanded combined AUD/AUD-CUD sample: C. Subtype 1. D. Subtype 2. All regions shown are significant at p-FDR<0.05. Color bar represents standardized beta weights.

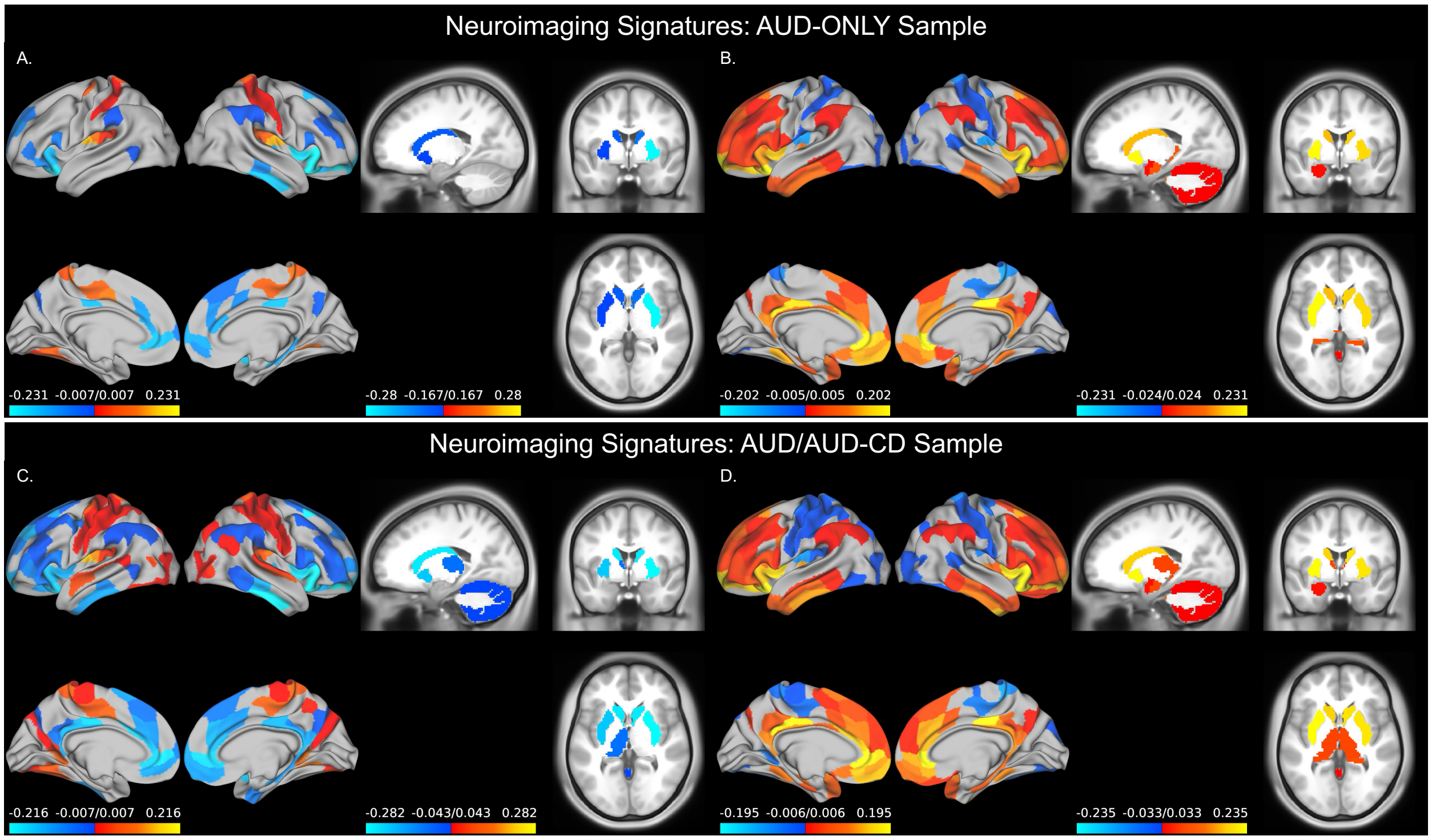

Supplementary Figure 11: Subtype Phenotypic and Clinical Characterization: Combined AUD/AUD-Cannabis Dependence Sample

Phenotypic and clinical characterization of the neuroimaging-derived HYDRA Subtypes in the expanded AUD/AUD-CD Sample. Extracted 18 phenotypic/clinical latent factors via Exploratory Factor Analysis from all the HCP measures (100 variables) and compared groups. Only factors demonstrating significant group differences are displayed. p-FDR **<.01; *** <.001.

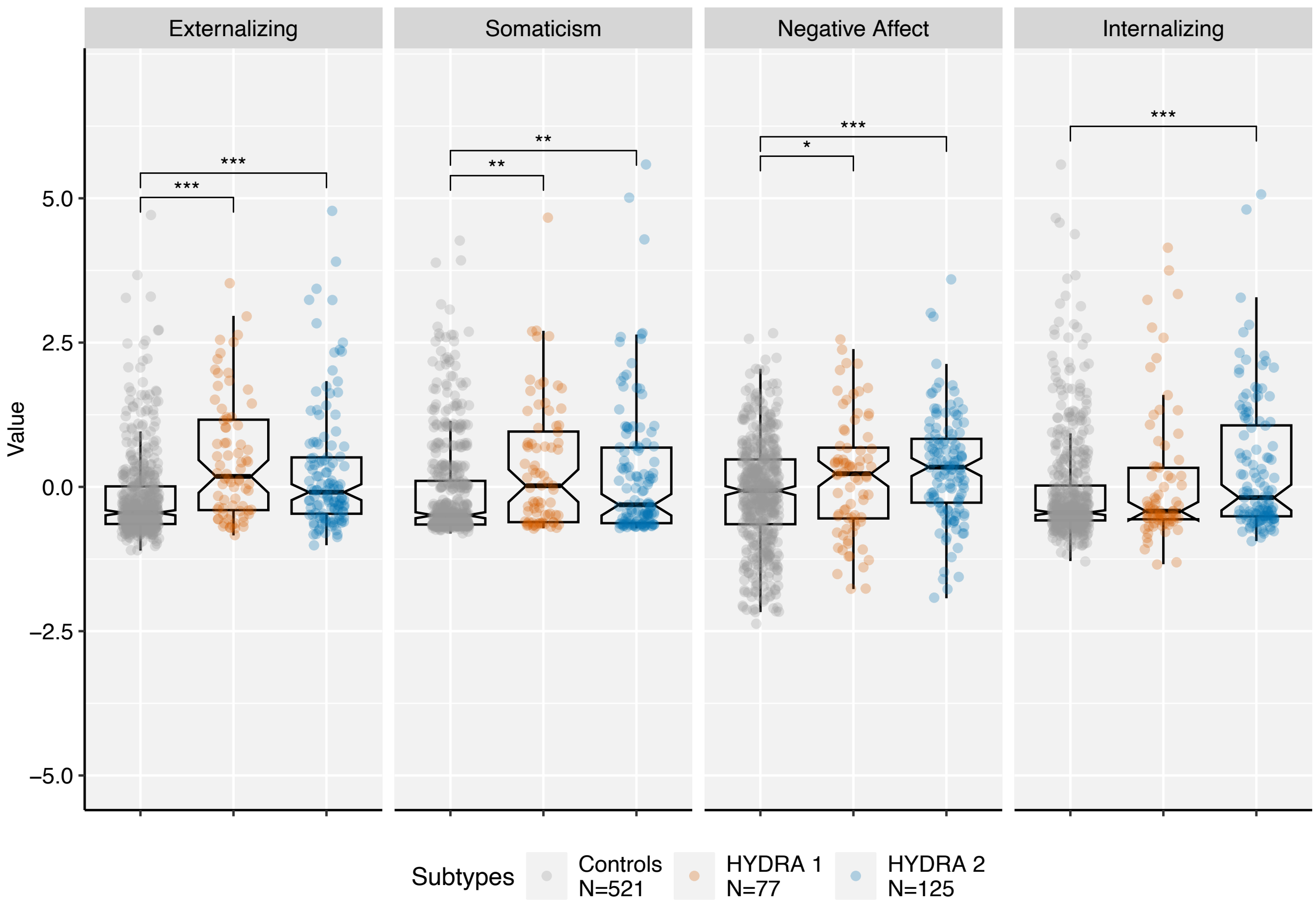

References

1. Bullmore ET, Bassett DS. Brain graphs: graphical models of the human brain connectome. Annu Rev Clin Psychol. 2011;7:113–40.

2. Garrison KA, Scheinost D, Finn ES, Shen X, Constable RT. The (in)stability of functional brain network measures across thresholds. Neuroimage. 2015 Sep;118:651–61.

3. Rubinov M, Sporns O. Complex network measures of brain connectivity: uses and interpretations. Neuroimage. 2010 Sep;52(3):1059–69.

4. van den Heuvel MP, Sporns O. Network hubs in the human brain. Trends Cogn Sci. 2013 Dec;17(12):683–96.

5. Latora V, Marchiori M. Efficient Behavior of Small-World Networks. Phys Rev Lett. 2001 Oct 17;87(19):198701.

6. Guimerà R, Nunes Amaral LA. Functional cartography of complex metabolic networks. Nature. 2005 Feb;433(7028):895–900.

7. Power JD, Schlaggar BL, Lessov-Schlaggar CN, Petersen SE. Evidence for hubs in human functional brain networks. Neuron. 2013 Aug 21;79(4):10.1016/j.neuron.2013.07.035.

8. Glasser MF, Coalson TS, Robinson EC, Hacker CD, Harwell J, Yacoub E, et al. A multi-modal parcellation of human cerebral cortex. Nature. 2016 Aug;536(7615):171–8.

9. Van Essen DC, Ugurbil K, Auerbach E, Barch D, Behrens TEJ, Bucholz R, et al. The Human Connectome Project: A data acquisition perspective. NeuroImage. 2012 Oct 1;62(4):2222–31.

10. Rawls E, Kummerfeld E, Zilverstand A. An integrated multimodal model of alcohol use disorder generated by data-driven causal discovery analysis. Commun Biol. 2021 Mar 31;4(1):1–12.

11. Glorfeld LW. An Improvement on Horn's Parallel Analysis Methodology for Selecting the Correct Number of Factors to Retain. Educational and Psychological Measurement. 1995 Jun 1;55(3):377–93.

12. Costello AB, Osborne J. Best practices in exploratory factor analysis: four recommendations for getting the most from your analysis. Practical Assessment, Research, and Evaluation. 2005;10:1-9.
